# Selective Semi‐Hydrogenation of Acetylene using a Single‐Atom Cobalt on Carbon Nitride Photocatalyst with Water as a Proton Source

**DOI:** 10.1002/smtd.202500527

**Published:** 2025-05-19

**Authors:** Anna Fortunato, Daniele Perilli, Alexandru Dron, Verónica Celorrio, Goran Dražić, Luka Ðorđević, Laura Calvillo, Cristiana Di Valentin, Francesca Arcudi

**Affiliations:** ^1^ Department of Chemical Sciences University of Padova Via F. Marzolo 1 Padova 35131 Italy; ^2^ Department of Materials Science Università degli Studi di Milano‐Bicocca Via Cozzi 55 Milano 20125 Italy; ^3^ Light Source Ltd, Diamond House Harwell Science and Innovation Campus Oxfordshire Didcot OX11 0DE UK; ^4^ Department of Materials Chemistry National Institute of Chemistry Hajdrihova 19 Ljubljana SI‐1000 Slovenia

**Keywords:** acetylene reduction, carbon nitride, ethylene, single‐atom catalyst

## Abstract

Light‐powered strategies for the semi‐hydrogenation of acetylene to ethylene are rapidly emerging as sustainable alternatives to the traditional thermochemical processes. The development of a robust, selective, as well as recyclable, non‐noble catalyst that can be powered by visible light and uses water as proton source to accomplish this important reaction remains a key challenge. Here the first demonstration of a cobalt single‐atom catalyst supported on carbon‐nitride (Co−CN) as an all‐in‐one photocatalyst for the semi‐hydrogenation of acetylene to ethylene is reported using water as the proton source, offering advantages over current hydrogenation technologies. Carbon nitride hosts the individual catalytic active sites of cobalt thus combining photosensitizer and cocatalyst in one unit, in line with first‐principles modelling. Under visible light irradiation, Co−CN reduces acetylene to ethylene with stable activity for over 40 days of continuous operation, ≥99.9% selectivity, and provides means for coupling organic upgrading to produce valuable oxidation products. The heterogeneous Co−CN can be easily recovered and reused repeatedly without loss of catalytic activity and structural integrity. Thereby, the integrated and recyclable platform overcomes the need of coupling a separate photosensitizer to a catalyst, and using noble metal catalysts with an external H_2_ gas feed.

## Graphical abstract

A sustainable alternative to the energy‐intensive traditional thermocatalytic hydrogenation of acetylene to ethylene is reported. The catalyst is a cobalt single‐atom catalyst supported on carbon nitride that is powered by visible light, uses water as a proton source and demonstrates high selectivity, stability, and recyclability.

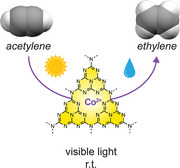

## 1 Introduction

**Figure 1:**
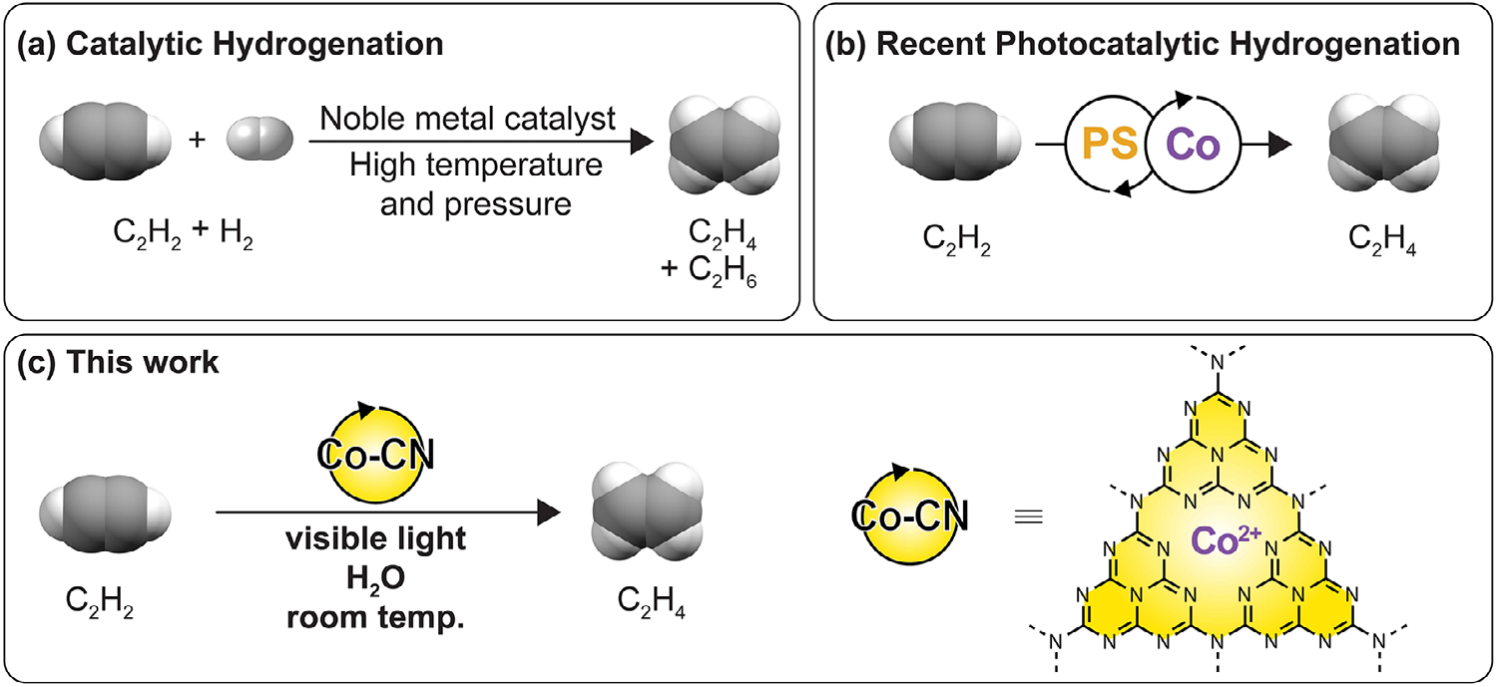
Semi‐hydrogenation of acetylene to ethylene: a) the state‐of‐the‐art process that uses noble‐metal catalysts and H_2_ at high temperature; b) recent photocatalytic hydrogenation routes that use cobalt catalysts in tandem with a photosensitizer and water or hydrogenated organics as proton donor at room temperature; c) this work that uses an all‐in‐one photocatalyst of single‐atom Co^2+^ sites within the cavity of carbon nitride (Co−CN) and water as proton donor at room temperature for the ≥99.9% selective production of ethylene.

Ethylene, a key commodity chemical and starting material for≈60% of all plastics,^[^ ^1^ ^]^ is commonly obtained by steam cracking together with acetylene impurities. Acetylene is poisonous to the Ziegler‐Natta polymerization catalysts and is therefore converted to ethylene to obtain polymer‐grade ethylene.^[^ ^2^, ^3^ ^]^ Traditional thermocatalytic routes for acetylene hydrogenation to ethylene, despite progress,^[^ ^4^, ^5^, ^6^ ^]^ still suffer from high temperatures (100–300 °C), H_2_ consumption, low selectivity for ethylene over ethane, and the need of a noble metal (Pd) catalyst (**Figure** **1**a).^[^ ^7^, ^8^, ^9^, ^10^, ^11^, ^12^ ^]^ This is a prime example of a system with ample room for improvement in terms of economics, sustainability, activity, and selectivity. Alternative routes to acetylene hydrogenation could also replace altogether the petroleum‐based ethylene production route, since acetylene can be produced commercially either from methane (from sustainable sources such as biogas) or calcium carbide.^[^ ^13^ ^]^

Recent efforts, including ours, have demonstrated acetylene hydrogenation catalysts powered either by electricity or light. Especially copper surfaces are used in the electrochemical field to reduce acetylene to ethylene.^[^ ^14^, ^15^, ^16^, ^17^ ^]^ Light‐powered catalysts are, at least, equally important since they can use directly sustainable and abundant solar energy. With 3d metals known to be active for the hydrogenation of alkyne substrates to alkenes either through transfer hydrogenation or by using molecular hydrogen,^[^ ^18^ ^]^ especially Co(I) states of cobalt catalysts have shown successful photochemical reduction of acetylene to ethylene.^[^ ^19^, ^20^, ^21^, ^22^, ^23^, ^24^ ^]^ We have contributed to this area of research reporting the first photocatalytic system for the semi‐hydrogenation of acetylene to ethylene that operates under visible light, uses water as the proton source and a non‐noble metal homogeneous cocatalyst (cobalt, in the form of cobalt porphyrin).^[^ ^19^ ^]^ Besides homogeneous,^[^ ^19^, ^22^, ^24^ ^]^ cobalt‐based heterogeneous catalysts in the form of metal‐organic frameworks (MOFs) or covalent‐organic frameworks (COFs) were also found to selectively reduce acetylene to ethylene when powered by light.^[^ ^20^, ^21^, ^23^, ^24^ ^]^ In this context, we have reported heterogeneous catalysts that incorporate unsaturated cobalt atoms into a porphyrin‐based or a cobaloxime‐based MOF. These cobalt heterogeneous catalysts were found to selectively reduce acetylene to ethylene and, as opposed to the homogeneous catalysts, could be recycled and reused several times. However, all these cobalt catalysts act only as cocatalysts and need a photosensitizer (e.g., tris(2,2′‐bipyridyl)ruthenium(II)) to be active in photocatalysis (Figure 1b). MOF catalysts were also found to undergo hydrolysis and degradation in aqueous solvents, and, therefore, the need to use acetonitrile and a hydrogenated organic as additional proton source.^[^ ^20^, ^24^ ^]^

Having shown that mononuclear metals are beneficial in heterogeneous catalytic hydrogenation,^[^ ^20^, ^24^ ^]^ we sought to explore a cobalt single‐atom catalyst supported on carbon nitride (Co−CN from now on) as an integrated heterogeneous photocatalyst for the semi‐hydrogenation of acetylene to ethylene in water (Figure 1c).

Although the concept of isolated atoms featuring unique reactivity^[^ ^25^ ^]^ has long existed in homogenous and enzymatic catalysis, the field of metal single‐atom heterogeneous catalysts is only recently establishing its unique position in bridging homogenous and heterogeneous catalysis, by bringing benefits from both fields.^[^ ^26^, ^27^ ^]^ Metal single‐atom catalysts have been applied in a wide range of chemical transformations, which include oxidations, hydrogenations, carbon‐carbon and carbon‐heteroatom couplings, hydroelementation, carboxylation, fluorination, cycloaddition reactions, oxygen or carbon dioxide or nitrogen reduction reactions, and oxygen or hydrogen evolution reactions.^[^ ^28^, ^29^, ^30^, ^31^, ^32^, ^33^, ^34^, ^35^ ^]^ In the context of electro‐ and photo‐catalytic hydrogenations, copper single‐atom catalysts supported either on titania^[^ ^36^ ^]^ or nitrogen‐doped carbon^[^ ^37^ ^]^ were shown to be active in the acetylene electroreduction, and palladium^[^ ^38^, ^39^ ^]^ or nickel^[^ ^40^, ^41^ ^]^ single‐atom catalysts supported on carbon nitride were shown to work for the photocatalytic hydrogenation of substituted alkenes and alkynes (e.g., styrene, 2‐ethynylnaphthalene) in water or water/organic solvent mixture.

Here, we utilize the strategy of anchoring single‐atom cobalt sites onto the carbon nitride surface to develop a fully heterogeneous photocatalytic system that uses water as a proton source for the selective semi‐hydrogenation of acetylene to ethylene. The Co−CN has the CN support that generates electron‐hole pairs under visible‐light irradiation and hosts the Co single‐atoms, thus effectively functioning as an all‐in‐one photocatalyst, in line with first‐principles modelling. We demonstrate that the Co−CN photocatalyst converts acetylene to ethylene in water with ≥99.9% selectivity for ethylene and retains its activity for 960 working hours. The heterogeneous nature of CN allows easy isolation of Co−CN, which can be recycled and reused five times without evidence of over‐hydrogenation or loss of activity.

## 2 Result and Discussion

Carbon nitride, an organic semiconductor, can be prepared from easily accessible and inexpensive precursors. Although initially the most studied host materials for single‐atom catalysts were metal oxides, tailored carbons have superseded all other hosts in the last years with carbon nitride being the most common organic semiconductor utilized.^[^ ^42^, ^43^, ^44^, ^45^, ^46^, ^47^ ^]^ In addition to an appropriate bandgap to generate electron‐hole pairs under visible light (≈2.7 eV), CN provides nitrogen‐rich cavities to accommodate and stabilize single‐atom catalysts.^[^ ^45^, ^48^, ^49^ ^]^ We synthetized and characterized the CN support and the single‐atom Co^2+^ sites within the cavity of CN to afford the Co−CN photocatalyst according to previously reported procedures (for details see the Supporting Information).^[^ ^50^ ^]^ In a first step, bulk CN was prepared by heating dicyanamide as source of carbon and nitrogen. After exfoliation through thermal treatment, the obtained CN sheets were dispersed in water with cobalt(II) chloride as metal precursor. This was followed by a reduction treatment and washing steps to obtain the Co−CN material, which was characterized through a combination of techniques. The X‐ray powder diffraction (XRD) pattern of Co−CN shows two distinct diffraction peaks at 2𝜃 equal to 12.8° and 27.5° corresponding to the (100) and (002) planes, respectively, of the graphite‐like carbon nitride system (Figure S1, Supporting Information) that match well with those reported in literature.^[^ ^50^, ^51^, ^52^ ^]^ The weak diffraction peak at 12.8° indicates an intralayer structural packing motif, specifically trigonal N linkage of tri‐*s*‐triazine units, while the intense sharp peak at 27.5° is due to the interplanar stacking of aromatic structures. The XRD diffraction pattern of Co−CN showed no additional peaks other than the characteristic ones of the metal‐free CN sheets, indicating that the structure of CN is well preserved after incorporation of the metal, and that no Co‐based species such as chlorides, nitrides or oxides as crystalline metal clusters or particles are present thereby suggesting the presence of atomic dispersion of Co atoms on the CN support (vide infra).

**Figure 2:**
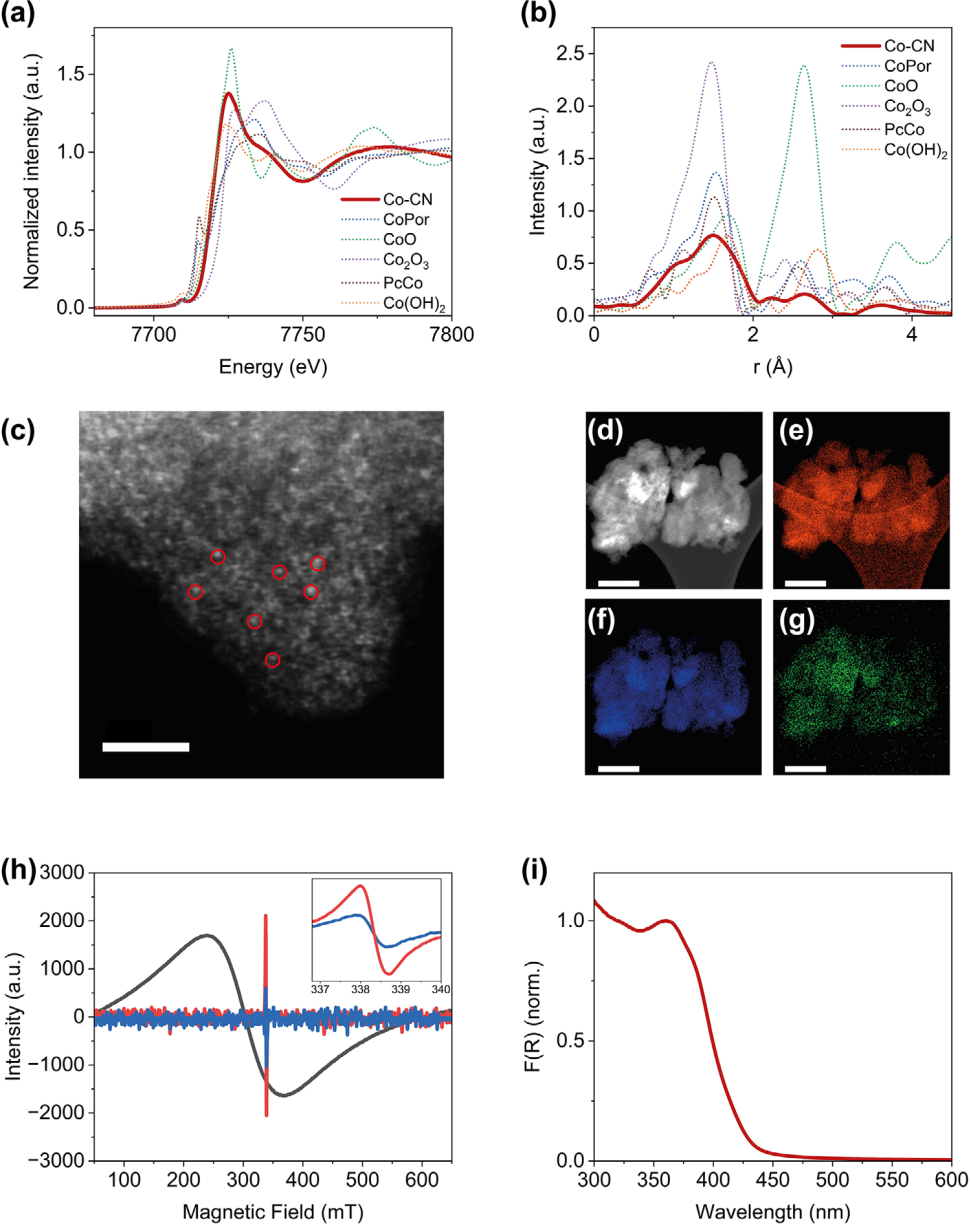
a) XANES region and (b) FT‐EXAFS spectra at the Co K edge of Co−CN (red) and of reference Co‐porphyrin (blue), CoO (green), Co_2_O_3_ (violet), Co‐phthalocyanine (purple), and Co(OH)_2_ (orange). c) HAADF‐STEM image of Co−CN, where cobalt single‐atoms are indicated with red circles. Scale bar 2 nm. d) DF‐STEM image of Co−CN and corresponding elemental mapping image (EDXS) of e) C kα, f) N kα and g) Co kα. Scale bar 300 nm. h) Solid state EPR spectra recorded at 77K of Co−CN (red), pristine CN (blue), and commercially available cobalt nanoparticles (cobalt(II, III) oxide, black). For Co−CN and pristine CN the signal intensity has been magnified 20 times (inset: zoom of the region of carbon radical 338 mT for Co−CN and pristine CN samples). i) (DR)UV–vis spectrum of Co−CN.

X‐ray photoelectron spectroscopy (XPS) reveals the presence of C, N, and Co elements, and allows for the surface chemical states of Co−CN to be analyzed (Figure S2, Supporting Information). The high‐resolution C 1*s* XPS spectrum (Table S1, Supporting Information) reveals a component at 288.3 eV, which is characteristic of the *sp* ^2^‐hybridized carbon (N−C═N) of the graphitic carbon nitride support.^[^ ^53^ ^]^ Two additional components at 284.7  and 286.2 eV are related to the adventitious carbon and its corresponding oxygen species, respectively. The N 1*s* region can be deconvoluted into three main peaks at 398.7, 399.9, and 401.15 eV, corresponding to pyridinic N (C═N−C), amino N ((C)_2_−NH), and graphitic N((C)_3_−N) species, respectively (Table S2, Supporting Information).^[^ ^54^ ^]^ The analysis of the N 1*s* region gives information about the CN structure and points to a high polymerization degree of a condensed heptazine‐based structure. A C/N molar ratio of 0.754 was estimated based on the XPS results, which is close to the theoretical value of 0.75.^[^ ^55^ ^]^ The Co 2*p* XPS region presents two peaks at 781.0 and 796.8 eV with their corresponding satellites, which are attributed to the Co 2*p*_3/2_ and Co 2*p_1_*_/2_ components of Co^2+^, respectively.^[^ ^56^, ^57^, ^58^, ^59^, ^60^, ^61^ ^]^ Consistent with XPS results, the X‐ray absorption near‐edge structure (XANES) of Co−CN indicates that Co is retained on the CN matrix as Co^2+^ species (**Figure** **2**a). The phase‐uncorrected Fourier‐transform (FT) extended X‐ray absorption fine structure (EXAFS) spectrum only shows one peak at 1.5 Å that can be ascribed to Co─N bonds (Figure 2b).^[^ ^62^, ^63^ ^]^ The best‐fit analysis result of EXAFS (Figure S3 and Table S3, Supporting Information) shows that each Co atom is bonded with three N atoms with an average Co−N distance of ≈2.07 Å. Contributions from Co−O species, such as residual hydration of the Co sites, could not be excluded. The absence of peaks in the region between 2.2 and 2.8 Å excludes, however, the presence of Co−Co or Co−O−Co clusters (Figure S4, Supporting Information), which strongly indicates the atomic dispersion of cobalt single‐atoms on the CN support, in agreement with XRD results.

The exclusive presence of single‐atom cobalt sites was confirmed by high‐angle annular dark‐field scanning transmission electron microscopy (HAADF‐STEM) and energy‐dispersive X‐ray spectroscopy (EDXS). No observable clusters or nanoparticles can be observed from the HAADF‐STEM images of the exfoliated sheet‐like structure of carbon nitride (Figure 2c–g), and EDXS shows individual Co atoms atomically dispersed and homogeneously distributed on the CN support (Figure 2g; Figure S5, Supporting Information). The absence of Co nanoparticles was further confirmed by electron paramagnetic resonance (EPR) measurements of the Co−CN powder as the typical broad EPR peak associated with ferromagnetic Co clusters or nanoparticles was not observed (Figure 2h).^[^ ^64^ ^]^ The EPR signal at *g* = 2.003 is ascribed to localized unpaired electrons hosted in the *p* orbital belonging to a *sp* ^2^ hybridized C atom (Figure 2h),^[^ ^65^, ^66^ ^]^ while the absence of a EPR signal from single Co^2+^ sites, even at 77K, could be attributed to a short relaxation time that is in agreement with previous reports.^[^ ^59^ ^]^

The optical properties of Co−CN were investigated using diffuse reflectance UV–vis ((DR)UV–vis) spectroscopy (Figure 2i). The material exhibits absorption in the visible region, with a calculated energy gap of 2.78 eV (Figure S6, Supporting Information) and a typical density of states of semiconductors (Figure S7, Supporting Information).

We evaluated the photocatalytic semi‐hydrogenation activity of a reaction mixture consisting of Co−CN photocatalyst and a sacrificial donor in pure water. In a typical experiment, 2.0 mL of the reaction mixture was illuminated under 1 atm of C_2_H_2_ using a 405 nm light‐emitting diode (LED, 140 mW∙cm^−2^). The details of our purging and photocatalysis setups are described elsewhere.^[^ ^19^ ^]^

After irradiation, the headspace was sampled and analyzed by a gas chromatograph (GC) equipped with both a thermal conductivity detector (TCD) and a flame ionization detector (FID), for details see Supporting Information. A typical gas chromatogram of the reaction mixture after irradiation shows conversion of acetylene to ethylene without appreciable over‐hydrogenation to ethane (Figures S8 and S9, Supporting Information).

The effect of different sacrificial donors on the photocatalytic conversion of C_2_H_2_ to C_2_H_4_ by the Co−CN system was evaluated, and the highest photocatalytic activity was achieved using triethanolamine (TEOA) (Figure S10, Supporting Information). The amount of C_2_H_4_ produced increased with TEOA concentration up to 2.0 m (Figure S11, Supporting Information), with the Co−CN catalyst showing stability under this condition in contrast to the previously reported heterogeneous Co‐based metal organic framework.^[^ ^20^ ^]^ The amount of C_2_H_4_ produced increased with the amount of Co−CN up to 10.0 mg (Figure S12, Supporting Information), with further increase in Co−CN quantity having a negligible effect.

**Figure 3:**
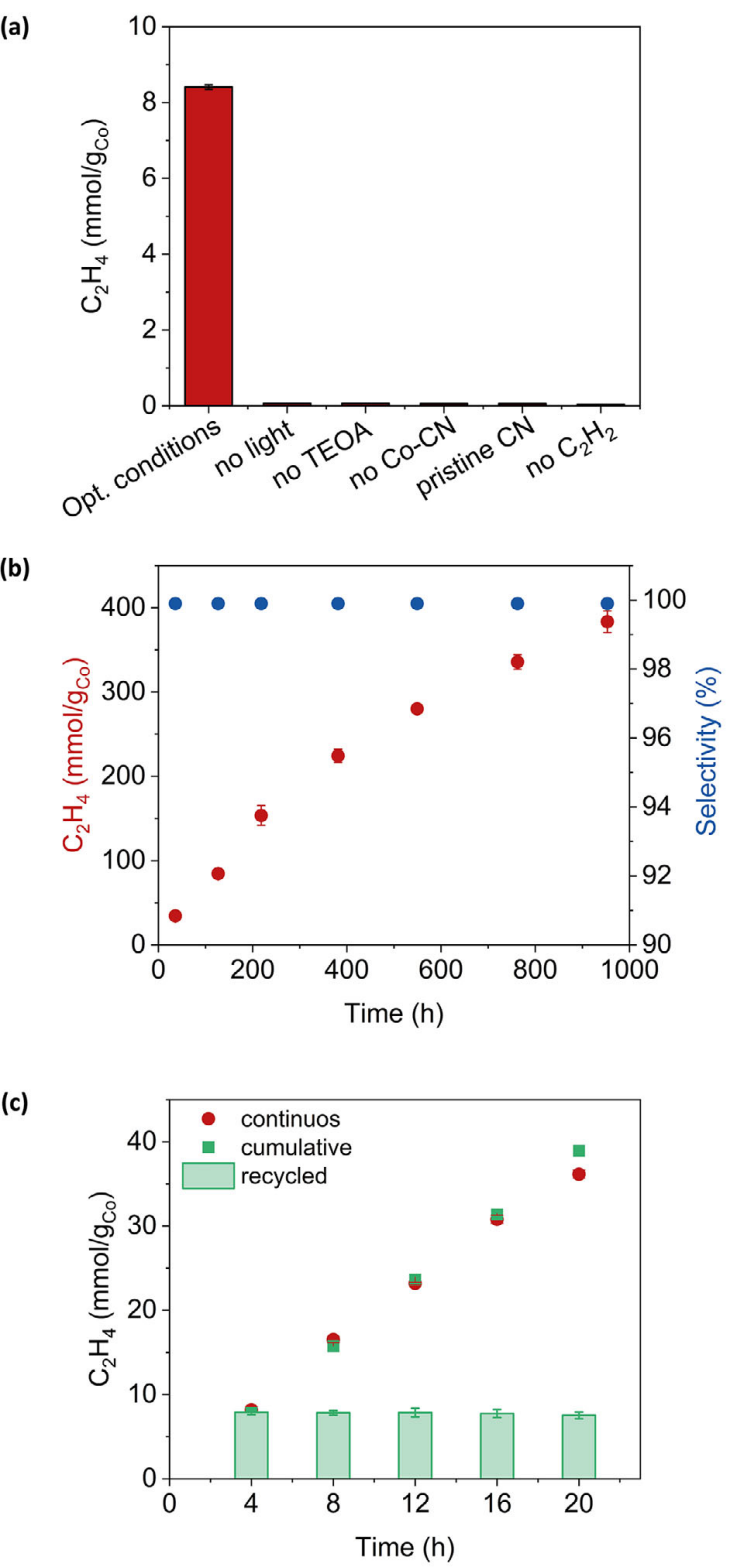
a) Ethylene production (mmol/g_Co_) by the Co−CN optimized system (labeled as optimized condition) containing 2.00 ± 0.05 mg Co−CN, 2.0 m TEOA in pure water at pH 10.3 ± 0.1 under C_2_H_2_ irradiated (405 nm, 140 mW∙cm^−2^) for 4 h, and for systems that differ from the optimized one, as indicated in the axis label. b) Ethylene production (mmol/g_Co_) and selectivity over ethane and hydrogen (%) as function of irradiation time for the Co−CN optimized system under C_2_H_2_ and irradiated at 405 nm (140 mW∙cm^−2^). c) Recyclability of the optimized Co−CN system under C_2_H_2_ and irradiated at 405 nm (140 mW∙cm^−2^). The red dots represent the ethylene production by the Co−CN optimized system during continuous irradiation for 20 h, green square histograms represent the ethylene evolved during 5 cycles of 4 h each, while the cumulative ethylene production for the recycling test is represented by the green dots. Error bars are calculated from two to three runs; uncertainty is ≤10%.

After optimizing the conditions, illumination for 4 h of a catalytic mixture containing 2.0 mg Co−CN and 2.0 m TEOA produced 8.4 mmol/g_Co_ of C_2_H_4_ and, importantly, no other gases, including H_2_ and C_2_H_6_, were detected (≥99.9% selectivity). The semi‐hydrogenation of C_2_H_2_ did not proceed in the absence of Co−CN, sacrificial donor, light or C_2_H_2_ feedstock, confirming the photocatalytic nature of the reaction (**Figure** **3**a). Notably, no C_2_H_4_ was produced in the presence of metal‐free carbon nitride as catalyst, indicating the pivotal role of Co in the reaction. Thus, Co−CN combines photosensitizer and cocatalyst in one unit, which we highlight once again as an important improvement over previously reported multicomponent systems^[^ ^19^, ^20^, ^21^, ^22^, ^23^, ^24^ ^]^ for acetylene photoreduction.

An important figure of merit of the CN‐based photocatalyst is its impressive stability and longevity. The catalyst remained productive over a period of 40 days (i.e., 960 h of illumination) without diminishing the ethylene production (Figure 3b). Over the entire illumination period, the catalytic mixture achieves an overall production of 390 mmol/g_Co_ of ethylene, with a ≥99.9% selectivity for ethylene over ethane and hydrogen. These results prove that our Co−CN is a robust, selective, and stable photocatalyst. Indeed, when we removed the Co−CN photocatalyst after 960 h of operation and illuminated the obtained supernatant, we observed no conversion of C_2_H_2_ to C_2_H_4_. This agrees with the inductively coupled plasma mass spectrometry (ICP‐MS) analysis (Table S4, Supporting Information) of the supernatant after prolonged irradiation detecting no Co leaching from the Co−CN photocatalyst. It is also important to note that, following 960 h of catalysis, the Co−CN showed no changes in XRD pattern (Figure S13, Supporting Information) and FT‐EXAFS profile (Figure S14, Supporting Information). No N_2_ evolution was observed after long irradiation of our photocatalytic system, suggesting the strong binding of N in the carbon nitride support.^[^ ^67^, ^68^ ^]^ As such, the post‐catalysis characterization demonstrates that the structural integrity of the Co−CN photocatalyst is entirely preserved even after a prolonged illumination period (40 days) of reducing efficiently and selectively acetylene to ethylene in water. Taken together, all these observations support the good photostability and durability of Co−CN under long‐term photocatalytic conditions.

The Co−CN powder allows for an easy and quantitative recovery of the photocatalyst from the reaction mixture by centrifugation. The recovered photocatalyst can be reused for further photocatalytic cycles to produce C_2_H_4_ without any loss of activity or selectivity, confirming again the good stability and durability of Co−CN. The total amount of ethylene produced in five recycling tests (4 h each) and the amount of ethylene produced during 20 h of continuous irradiation (Figure 3c) are in excellent agreement. As expected for a heterogeneous catalyst with a linear production of ethylene over time, the Co−CN catalyst can be efficiently recycled and reused multiple times. Post‐recycling, together with XRD, ICP‐MS and XAS measurements of Co−CN after 960 h of operation (Figures S13 and S14, Supporting Information) excluded chemical changes in the CN structure, Co leaching (Table S4, Supporting Information) or migration of Co atoms on the CN surface to form aggregates or nanoparticles thus confirming that Co remains on the CN structure as isolated atoms.

Beyond the semi‐hydrogenation of acetylene, we studied the photocatalyst's versatility demonstrating that it is competent for the conversion of phenylacetylene to styrene (Figure S15, Supporting Information). This indicates the applicability of Co−CN to other hydrogenation reactions.

Moreover, we studied the use of an organic substrate that yields value‐added oxidation products as electron donor, thus avoiding the undesirably waste of energy by sacrificial donors. Generally, the use of sacrificial donors is not ideal as they are considered waste.^[^ ^69^ ^]^ Coupling photocatalytic acetylene hydrogenation with organic upgrading to produce valuable reduction and oxidation products simultaneously has never been reported so far. Here, we could substitute the TEOA sacrificial donor with benzylamine. The illumination of a reaction mixture consisting of Co−CN and benzylamine under C_2_H_2_ produces C_2_H_4_ (Figure S10, Supporting Information) and *N*‐Benzylidenebenzylamine (Figure S16, Supporting Information) that is an important intermediate for the manufacture of fine chemicals, agrochemicals and pharmaceuticals.^[^ ^70^, ^71^, ^72^, ^73^ ^]^ This result demonstrates a win‐win strategy by coupling hydrogenation with oxidative organic synthesis in the same photocatalytic system, thus avoiding undesirably waste of energy of photogenerated holes and improving charge carrier utilization.

**Figure 4:**
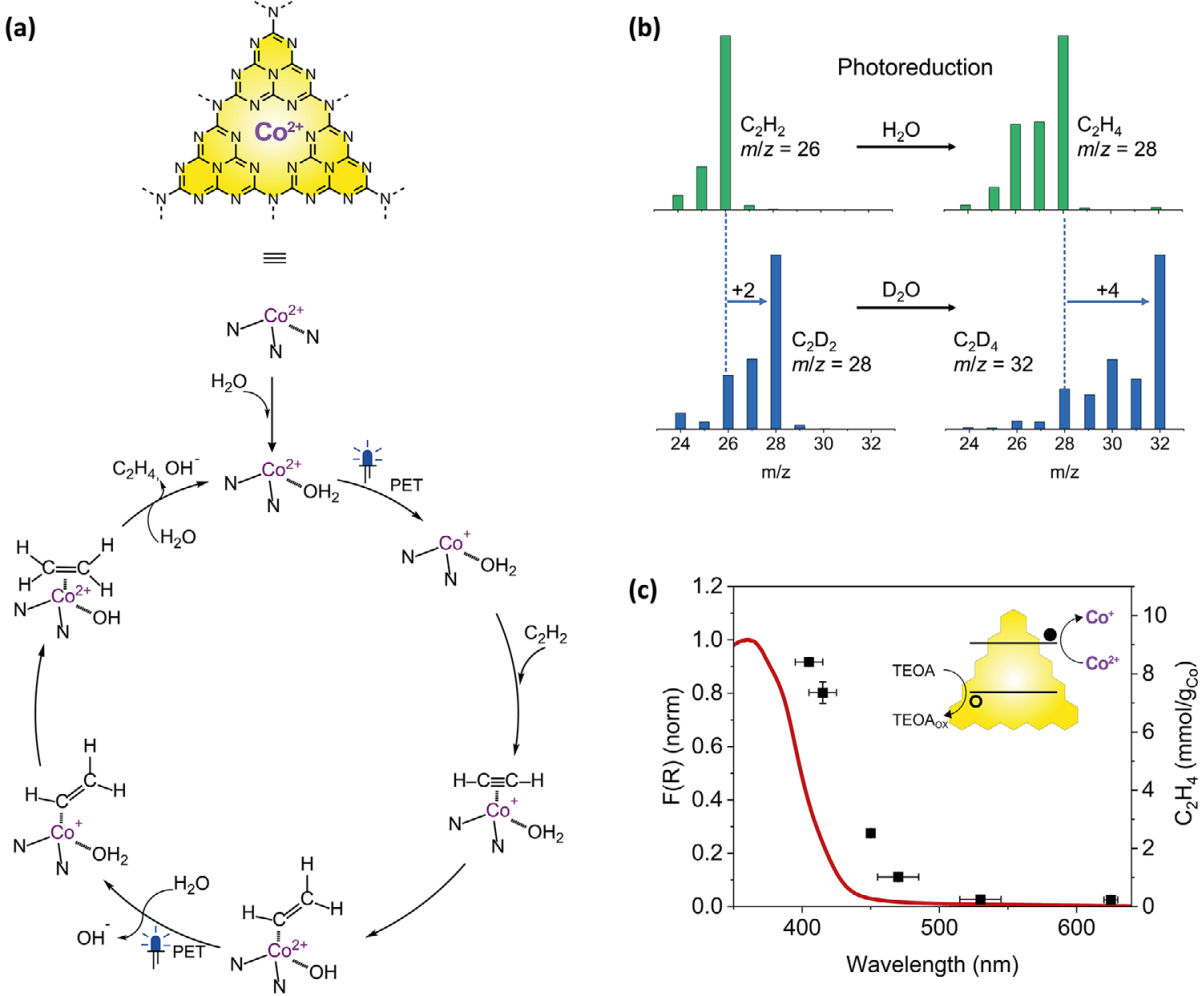
a) Proposed mechanism for the photoreduction of acetylene to ethylene by the cobalt single‐atoms anchored on the CN substrate through interaction with three N atoms, based on DFT calculations (see theoretical calculations, Figures S17 and S23 in Supporting Information). b) MS of acetylene (left) feedstock and ethylene (right) product for the irradiated (405 nm) Co−CN system under C_2_H_2_ in MilliQ H_2_O (green) or in D_2_O (blue). The shift m/z + 2 in H_2_O corresponds to incorporation of two hydrogens to produce C_2_H_4_ when starting with C_2_H_2_. The shifts m/z + 4 in D_2_O correspond to incorporation of two deuterium to produce C_2_D_4_ when starting with C_2_D_2_. c) Ethylene production by the Co−CN optimized system (mmol/g_Co_, right axis, black dots) containing 2.00 ± 0.05 mg Co−CN, 2.0 m TEOA in pure water at pH = 10.3 ± 0.1 under C_2_H_2_ and irradiated for 4 h as a function of irradiation wavelength (405, 415, 450, 470, 530, and 630 nm; 140 mW∙cm^−2^) overlaid on the absorption spectrum of Co−CN (left axis, red trace). Inset: representation of the internal photoinduced electron transfer process.

Based on experimental results and DFT calculations we proposed a mechanism, using a fully polymerized CN model from a previous work by some of us,^[^ ^74^ ^]^ for the photocatalytic semi‐hydrogenation of C_2_H_2_ using our Co−CN based system (**Figure** **4**a and S17, Supporting Information).

First, to shed light on the proton source, we conducted an isotope labelling experiment (Figure 4b). When the photoreduction was performed in H_2_O using C_2_H_2_ as feedstock, gas chromatography‐mass spectrometry (GC‐MS) revelated the formation of C_2_H_4_ (*m/*z = 28) as the only reaction product. This signal shifted to 32 when replacing H_2_O with D_2_O, which corresponds to the formation of C_2_D_4_ (*m*/*z* = 32) from C_2_D_2_ (*m*/*z* = 28), produced by the exchange between the feedstock C_2_H_2_ and D_2_O.^[^ ^75^, ^76^ ^]^ As a result, our experiments prove unambiguously that not only is acetylene the precursor for the observed C_2_H_4_, but also the protons added to make the C_2_H_4_ reduction product originate from water.

Both XANES and XPS investigations point to the presence of Co^2+^ species in our Co−CN photocatalyst. DFT calculations predict an exoergonic adsorption (ΔG = −2.65 eV) of water on a Co^2+^ that is originally coordinated to three pyridinic (pyr) N atoms (Figure S3, Supporting Information), leading to the formation of H_2_O−Co^2+^−2N(pyr) (Figure 4a and Figures S17 and S18, Supporting Information), which is adopted as the initial state of the photocatalytic cycle. The wavelength‐dependent production of ethylene closely matches the Co−CN absorption spectrum (Figure 4c), confirming the role of Co−CN as a true solo‐photocatalyst, operating both as light absorber and catalyst within the visible range. As the excitation wavelength increased from 405 to 630 nm, the production of C_2_H_4_ gradually dropped, confirming that the reducing electrons are derived from internal photoinduced electron transfer. Therefore, irradiation of H_2_O−Co^2+^−2N(pyr) generates electron/hole pairs and TEOA quenches the photogenerated holes whereas the photogenerated electrons reduces the Co^2+^ to Co^+^.^[^ ^19^, ^20^, ^24^, ^77^, ^78^ ^]^ Steady‐state photoluminescence (PL) spectra revealed that Co single‐atoms decrease PL intensity supporting the occurrence of electron transfer from CN to the Co atoms (Figure S19, Supporting Information).^[^ ^79^, ^80^ ^]^ Charge separation efficiency can be analyzed from the EPR spectrum during irradiation,^[^ ^66^ ^]^ while the recombination efficiency can be assessed by the decrease in EPR signals after switching off the light.^[^ ^81^ ^]^ As previously reported,^[^ ^82^ ^]^ the highest charge separation as well as the lowest recombination efficiency were observed for cobalt‐free CN compared to Co−CN (Figure S20, Supporting Information) suggesting that in Co−CN the photoinduced electrons are quickly transferred from the support to the cobalt species. Furthermore, the typical EPR signal of (2,2,6,6‐tetramethylpiperidine‐1‐yl)oxyl (TEMPO) was suppressed upon illumination of the reaction mixture under both acetylene or Ar atmosphere (Figure S21, Supporting Information). Indeed no ethylene was observed under the standard reaction condition in presence of TEMPO (Figure S22, Supporting Information) pointing to the critical role of photogenerated electrons.

On the thus formed low valent cobalt catalytic active species H_2_O−Co^+^−2N(pyr), DFT calculations predict a spontaneous adsorption of acetylene (ΔG = −0.63 eV). Coupled proton and electron transfer then occurs from the adsorbed water and the metal center, respectively, to the adsorbed acetylene as a result of water dissociation although with a free energy penalty of 0.55 eV (Figure 4a and Figure S23, Supporting Information). Our result also aligns with our previous work on the homogeneous porphyrin‐based system for acetylene semi‐hydrogenation,^[^ ^19^ ^]^ as well as with other studies on single‐atom catalysts for semi‐hydrogenation reactions,^[^ ^38^, ^40^ ^]^ where water dissociation and protonation of the π‐complex are the rate determining steps.^[^ ^83^ ^]^ A second photoexcitation and transfer of a proton was simulated by adding a photoexcited electron to Co^2+^ and introducing another water molecule. A similar coupled proton and electron transfer from the water and Co to the adsorbed *H−C═CH_2_ is predicted. Finally, calculations predict high thermodynamic energy cost for further hydrogenation of the C═C double bond (Figure S24, Supporting Information). In comparison, the desorption of ethylene is favored, closing the catalytic cycle and ensuring highly selective semi‐hydrogenation.

With the proposed mechanism, described above, as the most plausible from our experimental results and DFT calculations, we also considered alternative pathways. We could not rule out proton transfer from proximal protonated pyridinic N with a proton that could be transferred from the water molecule coordinated to the Co site (Figure S25a, Supporting Information) or from a non‐coordinate water molecule (Figure S25b, Supporting Information) to an uncoordinated proximal pyridinic nitrogen of the CN, thus forming a N−H bond. We note that, addition of pyrrole under standard reaction conditions led to a reduced ethylene production by half, which is ascribed to the possible role of the acidic pyrrole in blocking basic sites such as the pyridinic nitrogen (Figure S26, Supporting Information), in agreement with a previous report.^[^ ^40^ ^]^

The formation of a cobalt hydride species could be ruled out, as it is predicted to be an endoergonic process (ΔG = +1.47 eV) and therefore unlikely to form with our structural configuration^[^ ^84^ ^]^ and under the adopted working conditions. Furthermore, illumination of the reaction mixture under both acetylene or Ar atmosphere did not evolve any detectable H_2_ thus excluding that, for our system, acetylene semi‐reduction and H_2_ evolution proceed via the same catalytic intermediate with contribution from a cobalt hydride species.^[^ ^20^, ^24^ ^]^

## 3 Conclusion

In summary, we have demonstrated that a cobalt single‐atom catalyst supported on carbon nitride is competent for the visible light‐powered semi‐hydrogenation of acetylene to ethylene using water as the proton source. Developing photocatalytic systems that avoid the use of an external hydrogen feed and a noble metal catalyst provide powerful and sustainable alternatives to the state‐of‐the‐art Pd‐based thermocatalytic routes. The main advantages of the system reported herein compared to the recently (heterogeneous) photocatalytic systems^[^ ^20^, ^21^, ^23^, ^24^ ^]^ producing ethylene from acetylene are i) prominent selectivity, stability and longevity – operation for up to 960 h without losing activity and maintaining ≥99.9% selectivity for ethylene over ethane and H_2_, ii) easy recovery and reuse maintaining structural integrity and catalytic performance, iii) combine photosensitizer and cocatalyst in one unit as all‐in‐one photocatalyst, iv) use of water as proton source (and no other organic solvent or hydrogenated organic used), v) coupling semi‐hydrogenation of acetylene with oxidative organic synthesis thus avoiding undesirably waste of energy of photogenerated holes, as well as means to expand the scope with other alkynes.

Overall, this work demonstrates that cobalt single‐atom catalysts on photoactive supports hold great potential for this light‐powered reaction and could promote important steps forward replacing the traditional energy‐intensive and costly thermocatalytic semi‐hydrogenation of acetylene to ethylene and providing more sustainable industrial processes.

## Conflict of Interest

The authors declare no conflict of interest.

## Author Contributions

F.A. proposed and supervised the project. A.F., L.Ð., and F.A. designed the experiments. A.F. carried out experiments, with the help of A.D. for the preliminary investigations. V.C. and L.C. performed and analyzed XAS measurements. G.D. performed and analyzed TEM investigations. A.F. and L.Ð., designed, performed and analyzed EPR measurements. C.D.V. and D.P. designed, performed and analyzed first‐principles calculations. A.F. and F.A. wrote the manuscript. All authors were involved in manuscript revision and have approved the final version of the manuscript.

## Supporting information



Supporting Information

## Data Availability

The data that support the findings of this study are available from the corresponding author upon reasonable request.
