# Supplementary material for: Selective Semi‐Hydrogenation of Acetylene using a Single‐Atom Cobalt on Carbon Nitride Photocatalyst with Water as a Proton Source
Source: Small Methods. 2025 May 19;10(2):2500527. doi: 10.1002/smtd.202500527 (PMC12825346; doi:10.1002/smtd.202500527)
Supplement: Supplementary file 1 — Supporting Information [file SMTD-10-2500527-s001.pdf]

WILEY-VCH

Supporting Information

Selective Semi-Hydrogenation of Acetylene using a Single-Atom Cobalt on Carbon Nitride Photocatalyst with Water as a Proton Source

*Anna Fortunato, Daniele Perilli, Alexandru Dron, Verónica Celorrio, Goran Dražić, Luka Đorđević, Laura Calvillo, Cristiana Di Valentin, and Francesca Arcudi**

Table of Contents

Materials and Methods ... 3

- Safety warning ... 3
- Materials ... 3
- Photocatalytic reactions ... 4
- Chromatographic detection of gases ... 6
- Chromatographic detection of liquids ... 7
- UV-Vis Diffuse Reflectance Spectroscopy ... 8
- Photoluminescence Spectroscopy (PL) ... 8
- Powder X-Ray Diffraction (PXRD) ... 8
- X-Ray Photoelectron Spectroscopy (XPS) ... 8
- X-Ray Absorption Spectroscopy (XAS) ... 9
- Transmission Electron Microscopy (TEM) ... 9
- Theoretical calculations ... 9
- Inductively Coupled Plasma Mass Spectrometry (ICP-MS) ... 11
- Electron Paramagnetic Resonance (EPR) ... 12

Supplementary Figures ... 13

Supplementary Tables ... 39

Supplementary References ... 41

Materials and Methods

Safety warning

Acetylene is an extremely flammable gas. Buildup of acetylene vapors can result in fire or explosions if triggered by sparks. Acetylene may displace oxygen and cause rapid suffocation. In our experimental setup, the acetylene cylinder was fitted with a CGA 510 regulator equipped with a flashback arrestor and connected, through stainless steel tubing and a flow regulator, to a purging station that was placed inside a fume hood.[1] A Snoop® solution was applied to fittings and joints to inspect for leaks, until no bubble formation was observed. There was no electrical equipment in the fume hood.

Materials

Dicyandiamide (Alfa Aesar, 99%), cobalt chloride hexahydrate ($CoCl_2·6H_2O$, Alfa Aesar, 98%), sodium borohydride ($NaBH_4$, Sigma-Aldrich, ≥98%), triethanolamine (TEOA, Sigma-Aldrich, ≥99.0%), sodium ascorbate (NaAsc, Sigma-Aldrich, ≥98.0%), methanol (MeOH, Supelco, max. 0.003% H_2O), benzylamine (Sigma-Aldrich, ≥99.0%), ethylenediaminetetracetic acid (EDTA, VWR, ≥99.5%), cobalt nanoparticles (cobalt(II,III) oxide, 99.5%, Sigma-Aldrich), 4-hydroxy-2,2,6,6-tetramethylpiperidine 1-oxyl, (4-hydroxy-TEMPO, Sigma-Aldrich) phenylacetylene (Sigma-Aldrich, 98%), styrene (Sigma-Aldrich), and D_2O (Sigma-Aldrich, 99.9% atom D) were used as received.

Ultrapure water (MilliQ, >18.3 MΩ cm) was obtained using a PURELAB® flex 4system.

Synthesis of 2-(1,3-dimethyl-2,3-dihydro-1H-benzimidazol-2-yl)benzoic acid (BI(CO_2H)H).

BI(CO_2H)H was synthesized according to a published procedure.[2]

Synthesis of carbon nitride.

The synthesis of CN was performed following a literature procedure.[3] Dicyandiamide was used as the source of carbon and nitrogen. In a typical experiment, 5.0 g of dicyandiamide was

spread evenly on an alumina crucible and heated in air at 550 °C (heating rate, 10 °C min^{-1}) for 3 h. The obtained yellow solid was grinded, evenly spread on an alumina crucible and heated at 520 °C (heating rate, 2 °C min^{-1}) for 4.5 h. A pale-yellow powder was obtained (4.5 g). This sample, denoted as CN, was used as photoactive support for the dispersion of the metal species. The steps detailed above afforded a host material with structural features (see characterization in the main text and SI) that well compare with those in literature.[3]

Synthesis of Co−CN.

Co single-atoms were dispersed on the CN host adapting a literature procedure for nickel-doped carbon nitride.[3] 250 mg of CN are dispersed in 3 mL of MilliQ water. The $CoCl_2{\cdot}6H_2O$ metal precursor (0.42 mol) was added dropwise to the CN dispersion. The mixture was kept under sonication for 30 min and then stirred for 12 h at room temperature. Then, 750 mg of sodium borohydride was carefully added, and the reaction mixture was further stirred at 80 °C for 8 h. The solid was then collected by centrifugation. A 0.1 M TEOA solution (15 mL) was added, and the mixture was stirred for 12 h at room temperature. Finally, the sample was collected by centrifugation, washed three times with MilliQ water (20 mL) and dried overnight under vacuum obtaining 300 mg of Co−CN. With the steps and modifications from the literature procedure[3] detailed above, it was observed the exclusive presence of cobalt as single-sites.

Photocatalytic reactions

Samples were prepared in a 9.0 mL screw cap vial (B7800-3, Thermo Scientific) equipped with a micro stir bar (10 mm, Fisher Scientific) and sealed with silicone/PTFE septum (TS-12713, Thermo Scientific) and cap (open top, TS-13216, Thermo Scientific).

Vials for the semi-hydrogenation of acetylene were charged with 2.00 ± 0.05 mg of catalyst, which was dispersed in 2.0 mL of a water solution containing the sacrificial donor. The vials were sealed and purged either for 10 minutes with Ar (or He) followed by 5 minutes with C_2H_2 (≥99.5 vol.%, Airgas) or (ii) 15 minutes with Ar by using steel needles inserted through the

septum as inlet (inserted into the solution) and outlet (venting the headspace to the surrounding atmosphere). After purging for the time specified, the pressure of the headspace was then equilibrated to 1 atm. The vials were then illuminated (irradiated area 1.767 cm^2) using a homebuilt photoreactor made of 405 nm (U60 (400-410 nm)) LEDs (High Power LED Star, LEDsupply.com) with a light intensity of 140 $mW \cdot cm^{-2}$ (measured using an Optical Power Meter PM100D with Optical Sensor S120VC from Thorlabs). Each vial was suspended on top of a single LED, equipped with a lens, using a homebuilt sample holder. The vials were continuously stirred at 500 rpm during irradiation.

Vials for the semi-hydrogenation of phenylacetylene were charged with 2.00 ± 0.05 mg of catalyst and phenylacetylene (10 mM). To the mixture, a TEOA (1 M) solution in acetonitrile was added reaching a final volume of 2.0 mL, and then the vials were sealed and purged for 15 minutes with Ar by using steel needles inserted through the septum as inlet (inserted into the solution) and outlet (venting the headspace to the surrounding atmosphere). The vials were then illuminated (irradiated area 1.767 cm^2) using a homebuilt photoreactor made of 405 nm (U60 (400-410 nm)) LEDs (High Power LED Star, LEDsupply.com) with a light intensity of 140 $mW \cdot cm^{-2}$ (measured using an Optical Power Meter PM100D with Optical Sensor S120VC from Thorlabs). Each vial was suspended on top of a single LED, equipped with a lens, using a homebuilt sample holder. The vials were continuously stirred at 500 rpm during irradiation.

The samples of Figure 4c were charged with 2.00 ± 0.05 mg of catalyst, which was dispersed in 2.0 mL of a water solution containing 2 M TEOA. The vials were sealed and purged for 10 minutes with Ar followed by 5 minutes with C_2H_2 (≥99.5 vol.%, Airgas) by using steel needles inserted through the septum as inlet (inserted into the solution) and outlet (venting the headspace to the surrounding atmosphere). After purging for the time specified, the pressure of the headspace was then equilibrated to 1 atm. The vials were then illuminated (irradiated area 1.767 cm^2) using a homebuilt photoreactor made of 405 nm, 415 nm, 450 nm, 470 nm, 530 nm, or 630 nm LEDs (High Power LED Star, LEDsupply.com) with a light intensity of 140

mW·cm^{-2} (measured using an Optical Power Meter PM100D with Optical Sensor S120VC from Thorlabs). Each vial was suspended on top of a single LED, equipped with a lens, using a homebuilt sample holder. The vials were continuously stirred at 500 rpm during irradiation.

The samples for the isotope labelling experiment (Figure 4b) were charged with 2.00 ± 0.05 mg of catalyst, which was dispersed in 2.0 mL of a D_2O solution containing 2 M TEOA. The vials were sealed and purged for 10 minutes with Ar followed by 5 minutes with C_2H_2 (≥99.5 vol.%, Airgas) by using steel needles inserted through the septum as inlet (inserted into the solution) and outlet (venting the headspace to the surrounding atmosphere). After purging for the time specified, the pressure of the headspace was then equilibrated to 1 atm. The vials were then illuminated (irradiated area 1.767 cm^2) using a homebuilt photoreactor made of 405 nm (U60 (400-410 nm)) LEDs (High Power LED Star, LEDsupply.com) with a light intensity of 140 mW·cm^{-2} (measured using an Optical Power Meter PM100D with Optical Sensor S120VC from Thorlabs). Each vial was suspended on top of a single LED, equipped with a lens, using a homebuilt sample holder. The vials were continuously stirred at 500 rpm during irradiation.

<u>Chromatographic detection of gases</u>

GC-MS experiments were performed on an Agilent Technologies 7890A GC system coupled with a 5975C VL MSD with Triple-Axis Detector. The GC was equipped with a HPPLOT Q column, the inlet temperature was 200 °C, the He carrier gas flow was 1 mL·min^{-1} at a pressure of 4.1 psi. For the detection of ethylene, acetylene, and ethane the oven temperature was kept at 110 °C for 6.5 min, and then heated to 250 °C using a 15 °C·min^{-1} ramp (total run time 18.8 min). Headspace samples were manually injected using gas-tight Hamilton syringes (100 μL).

For the detection and quantification of acetylene and ethane in the photoreduction, GC experiments were performed on an Agilent Technologies 8860 GC system coupled with flame ionization detector (FID) and a thermal conductivity (TCD) detector. The system was equipped with a HP-PLOT U and a MS-5A column, Ar carrier gas flow was 6 mL·min^{-1} at a constant

pressure of 11.121 psi, the FID and TCD detectors were kept at 250 °C. For the detection of ethylene, acetylene, and ethane the oven temperature was kept at 40 °C for 5 min, then heated to 110 °C using a 20 °C·min^{-1} ramp, and kept at 110 °C for 9 min (total run time 17.5 min). Headspace samples were injected using PAL3 series 2 Autosampler Systems equipped with a gas-tight syringe (SGE autosampler syringe) injecting 100 μL.

Calibration curves for C_2H_4, C_2H_6 and H_2 were collected by injecting known quantities of gas mixture standards containing C_2H_4 (99.95 vol.% or 2.5 vol.% or 30 vol.%), C_2H_6 (2.5 vol.%), or H_2 (99.95 vol.% or 2.5 vol.%). All calibration curves are reported in Figure S9. Injections were performed either using gas-tight Hamilton syringes (10 − 100 μL) or using PAL3 series 2 Autosampler System equipped with a gas-tight syringe (SGE autosampler syringe) injecting 100 μL. Calibration injections were performed at least in triplicate.

The selectivity for ethylene (S_{C2H4}) for the photoreduction of acetylene is calculated as follows:

$$S_{C2H4}\,(\%) = \frac{mol_{C_2H_4}}{mol_{C_2H_4} + mol_{C_2H_6} + mol_{H_2}} \times 100$$

Selectivity for C_2H_4 is reported as ≥99.9% when no quantifiable C_2H_6 and H_2 are detected in the gas chromatograms. Experiments were performed at least in duplicate.

Chromatographic detection of liquids

GC-MS experiments were performed on an Agilent Technologies 6850 Network GC system coupled with a 5975 Mass Selective Detector. The GC was equipped with a HP-5ms column, the inlet temperature was 220 °C, the He carrier gas flow was 1.0 mL·min^{-1} at a pressure of 7.7 psi. For the detection of liquids, the oven temperature was kept at 50 °C for 3 min, and then heated to 290 °C using a 20 °C·min^{-1} ramp, and kept at 290 °C for 3 min (total run time 18 min).

For the photoreduction of C_2H_2 in presence of benzylamine, the solution was extracted with DCM thrice, dried over $MgSO_4$, removed under reduced pressure and then the product (50 uL) was diluted in acetonitrile (1.95 mL).

UV-Vis Diffuse Reflectance Spectroscopy

Diffuse reflectance UV-Vis spectra were recorded on a Schimadzu UV-2600i equipped with an ISR-2600Plus integrating sphere. Ultrafine $BaSO_4$ powder was used as a reference. The powders of the samples were placed in the holder up to ca. 3 cm diameter and 5 mm depth. The Kubelka-Munk function was applied to all spectra.

Photoluminescence Spectroscopy (PL)

Solid steady-state photoluminescence spectra were recorded on an FLS1000 Instrument (Edinburgh Instruments) equipped with an integrating sphere coated with $BaSO_4$. The powders of the samples were placed in a holder up to ca.1 cm diameter and 2 mm depth.

Powder X-Ray Diffraction (PXRD)

PXRD measurements were collected on a Bruker D8 Advance instrument operating with a Cu K_α (λ = 1.54 Å) radiation source generated at 40 kV and 40 mA. Samples were prepared by packing the Co–CN powder into metallic flat disc transmission holders.

X-Ray Photoelectron Spectroscopy (XPS)

XPS analyses were performed using a Thermo Scientific ESCALAB QXi spectrometer with a monochromatized Al Kα source (hν = 1486.68 eV). High resolution XPS spectra were obtained using a 20 eV pass energy and 0.1 eV steps. The XPS spectra were analysed using the XPSPeak4.1 software.

X-Ray Absorption Spectroscopy (XAS)

XAS measurements were recorded on beamline B18 at the Diamond Light Source (UK) with ring energy of 3 GeV and a current of 300 mA. The monochromator used was Si(311) crystals operating in Quick EXAFS (QEXAFS) mode. Pellets of Co−CN and of the references were measured in transmission and fluorescence modes at the Co K absorption edge and at 298 K using a 36-element Ge detector. The Co foil was measured simultaneously. The cycled Co–CN and the post photocatalysis samples were also measured by *ex situ* XAS in fluorescence mode. The Athena software was used to process and analyze the data of EXAFS and XANES.

Transmission Electron Microscopy (TEM)

TEM images were acquired with an FEI Tecnai G12 microscope. High resolution STEM analyses were performed on a probe Cs-corrected Scanning Transmission Electron Microscope, JEOL ARM 200 CF, operated at 80 keV to reduce the impact of the electron beam on the sample. The probe semi-convergence angle was 24 mrad and the HAADF detection angles were set to 68–185 mrad. For chemical analysis a Jeol Centurio EDXS system with a 100 mm^2 SDD detector and Gatan Quantum ER double EELS spectrometer were used.

Theoretical calculations

a) Computational details

Density Functional Theory (DFT) calculations were performed using the plane-wave-based Quantum ESPRESSO (QE) package to compute the free energy profiles,[4, 5] and the CRYSTAL17 (CRY17) package to evaluate the electronic structure properties.[6] In the QE calculations, ultrasoft pseudopotentials[7] were employed to describe electron-ion interactions, treating H (1*s*), C (2*s*, 2*p*), N (2*s*, 2*p*), O (2*s*, 2*p*), and Co (3*d*, 4*s*) as valence electrons. Energy cutoffs of 46 Ry for kinetic energy and 326 Ry for charge density expansion were used throughout. Van der Waals interactions were accounted for using the DFT-D3 formalism.[8] To

properly describe the *d*-electrons, a Hubbard U correction[9] of 3.0 eV was applied to Co, in agreement with previous studies. Spin polarization was included in all calculations.

For the CRY17 calculations, a Gaussian-type localized basis set was used in conjunction with the hybrid HSE06 functional[10] and D3[8] dispersion correction. The Kohn–Sham orbitals were expanded using the all-electron POB-TZVP-rev2[11] basis set for all atoms in the system (C, N, and Co). Spin polarization was included in all calculations involving Co species. Geometry optimizations were carried out using a 2 × 2 × 1 k-point mesh, while the density of states (DOS) and projected density of states (PDOS) were computed using a 12 × 12 × 1 k-point mesh, with the vacuum level set as the zero-energy reference. Spin polarization was included in calculations with Co.

All energy (both SCF and geometry optimizations) and force convergence thresholds were kept at their respective default values in the software packages used.

b) Computational model of Co-CN

For the simulation of the CN system, based on experimental results, we employed a fully polymerized periodic model, in which tri-s-triazine (heptazine) units are interconnected by nitrogen atoms, forming an extended polymeric structure. As previously proposed by some of us,[12] the unit cell consists of four heptazine units (highlighted in purple in Figure S3), providing greater structural flexibility, four atomic voids and resulting in a non-planar configuration. In one of the voids, we then introduced one Co^{2+} ion, in agreement with XPS experiments, which, after atomic optimization, results to be coordinated by three pyridinic nitrogen atoms (Figure S3). A second Co^{2+} ion, put in a different void, is used in the DFT calculations to balance overall atomic charges along the catalytic cycle. The atomic charge was verified via Bader charge analysis[13] and by spin configuration analysis. Notably, Co^{2+} exhibited a high-spin configuration with three unpaired *d* electrons.

Geometry relaxations were performed using a 2 × 2 × 1 Monkhorst-Pack[14] k-points mesh. To minimize interactions between adjacent periodic images, a vacuum space of approximately 21 Å was introduced perpendicular to the surface.

c) Computational thermochemistry

The free energies (G) of the intermediates (at T = 298.15 K) were computed by correcting the electronic energies (E) for the zero-point energy (ZPE), heat capacity (∫Cp dT), and the vibrational entropic term (S_{vib}), calculated as follows:

$$G = E + ZPE + \int C_p \, dT - TS_{vib}$$

Vibrational modes were determined via normal-mode analysis, considering all 3N degrees of freedom of the adsorbates (reactants and reaction intermediates) and Co^{2+} bonded to adsorbates within the harmonic oscillator approximation. ZPE corrections were included by computing and diagonalizing the dynamical matrix at the Γ-point.

For gas-phase C_2H_2, C_2H_4, and C_2H_6, the ideal gas limit approximation was applied, assuming a pressure of 1 bar. In contrast, the entropy of gas-phase water was calculated at 0.035 bar, corresponding to its equilibrium pressure in contact with liquid water at 298 K. Under these conditions, the free energy of gas-phase water is equivalent to that of liquid water.[15, 16]

Inductively Coupled Plasma Mass Spectrometry (ICP-MS)

The ICP-MS measurements were performed on a Agilent 7700x ICP-MS (Agilent Technologies International Japan, Ltd., Tokyo, Japan). The instrument features an octupole collision cell operating in kinetic energy discrimination mode, effectively removing polyatomic and argon-based interferences. For each Co–CN sample, 2 mg (with exact mass measured using an analytical balance) were digested with 0.75 g of nitric acid in a 15 mL falcon and heated at 100 °C for 2 h. The resulting solution was diluted to a final mass of 14 g using MilliQ water. For the surnatant, 0.5 g of the solution were added in a 15 mL falcon along with 0.75 g of nitric

acid. The falcon was then heated at 100 °C for 2 h and then diluted to a final mass of 15 g using MilliQ water.

Electron Paramagnetic Resonance (EPR)

EPR spectra were acquired on a Bruker ESR5000 operating at a microwave frequency of 9.46 GHz. The powders were transferred to an EPR tube (4 mm). The EPR spectra were recorded using ESRStudio both at room temperature and at 77 K by freezing the tube in liquid N_2 with 5.0 mT microwave power, 1.0 mT modulation and 120 seconds sweep time, and finally baseline-corrected.

Supplementary Figures

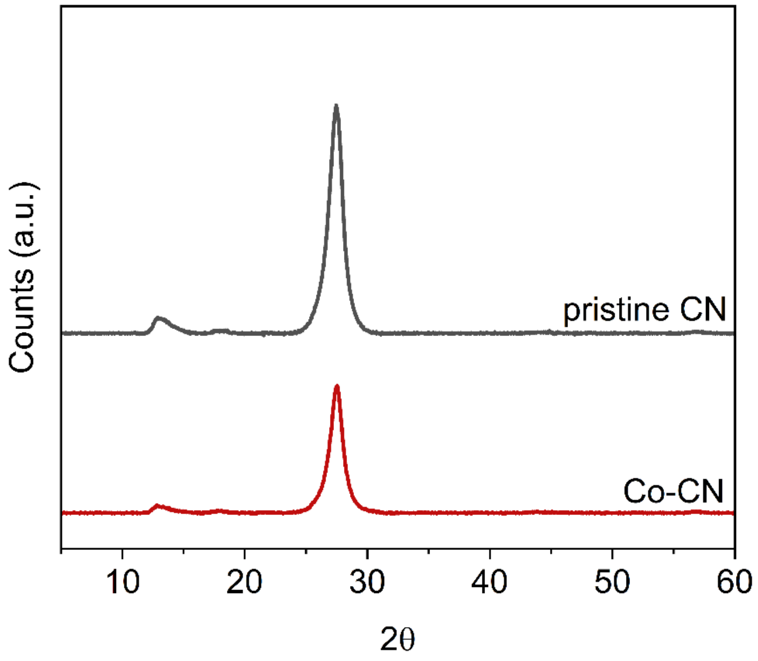

Figure S1. PXRD patterns of pristine CN (black) and Co−CN (red) samples.

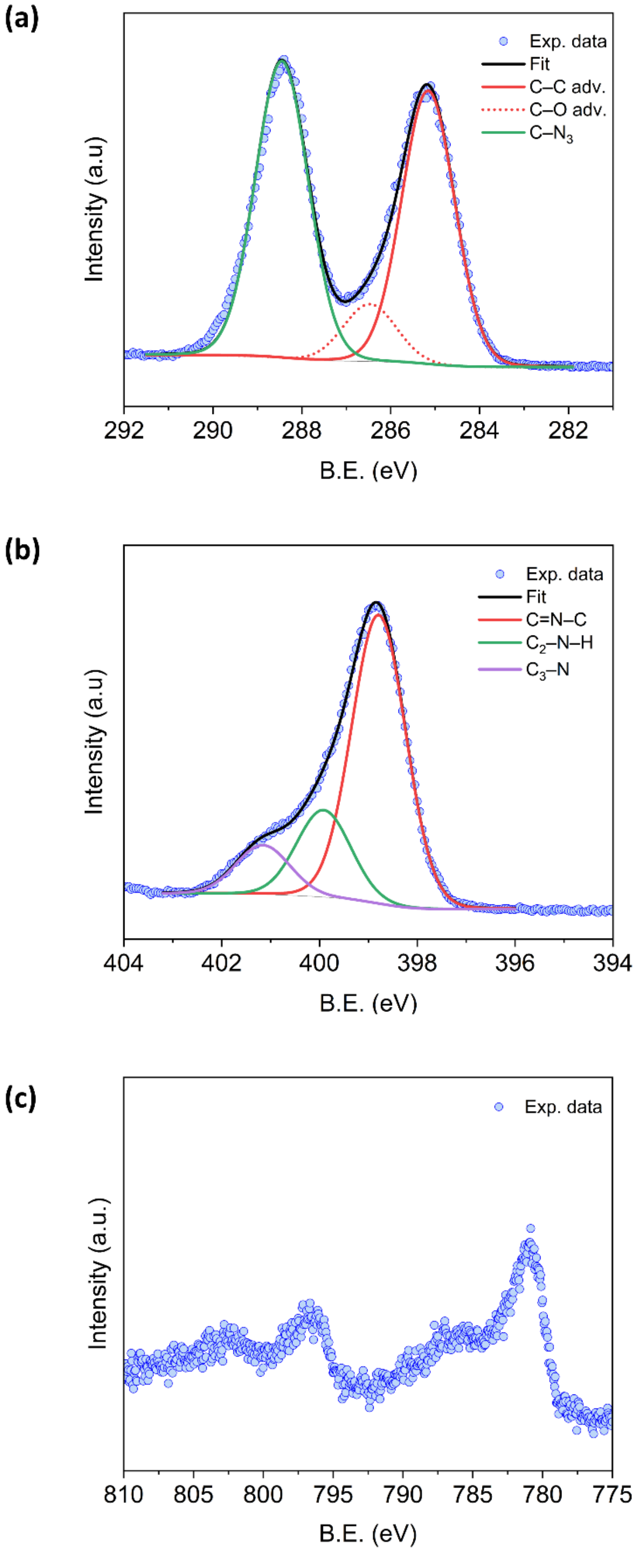

Figure S2. XPS spectra of Co−CN (a) C 1*s*, (b) N 1*s* and (c) Co 2*p*.

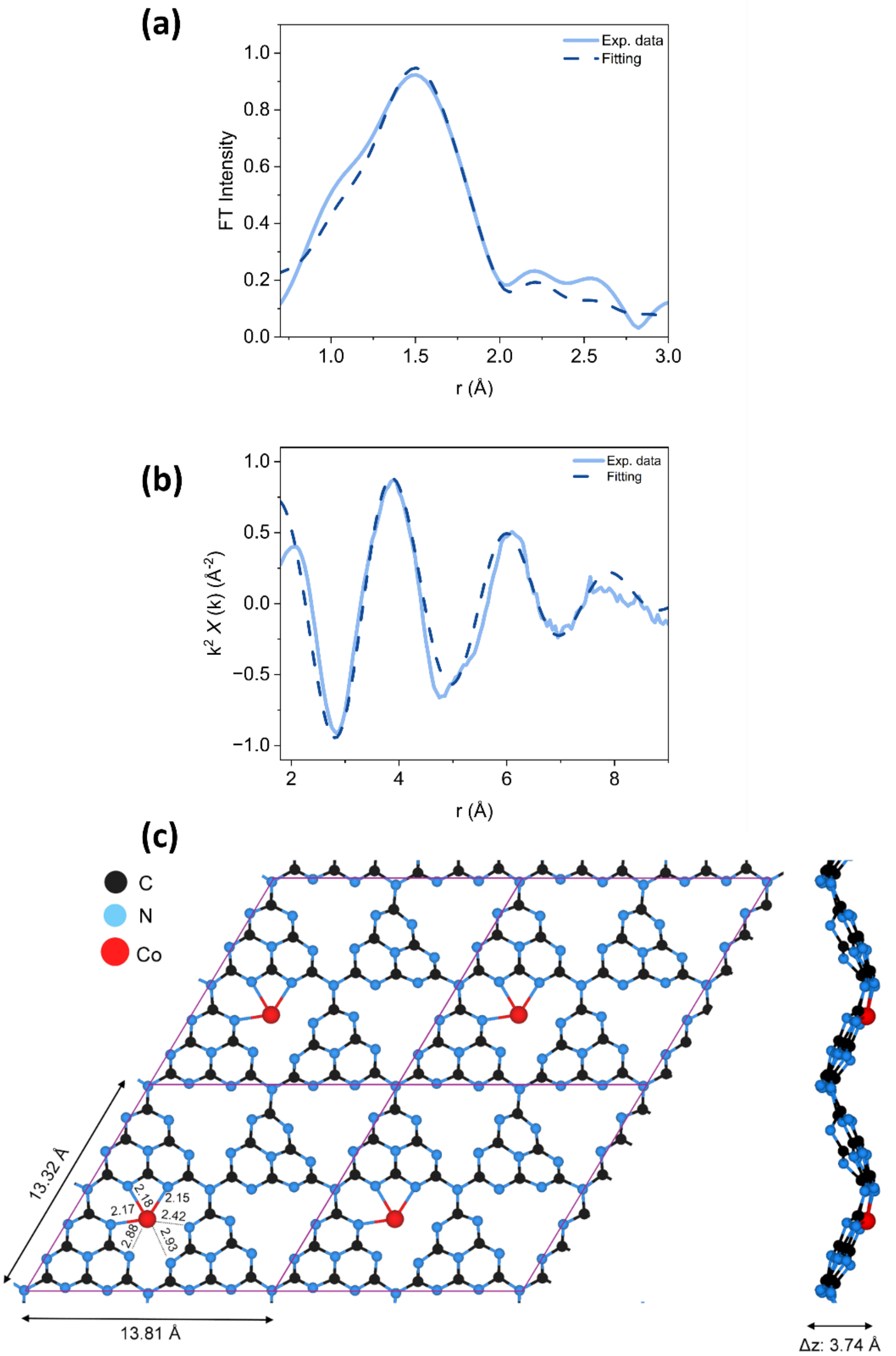

Figure S3. (a) Magnitude and (b) imaginary parts of the k^2-weighted phase-uncorrected FT-EXAFS extracted in the 2.1-12 Å^{-1} k range of Co–CN (solid line) and the corresponding best fits in R-space in the range 1.0-2.5 Å. (c) ball-and-stick representation (top and side views) of the Co–CN model. The supercell is outlined in purple, with cell parameters provided in Å. The side view includes the corrugation amplitude (Δz in Å). Atom colors: carbon (black), nitrogen (light blue), and cobalt (red). Co–N distances are given in Å, with those exceeding 2.2 Å represented by dashed lines. The Co metal center is coordinated by three pyridinic N.

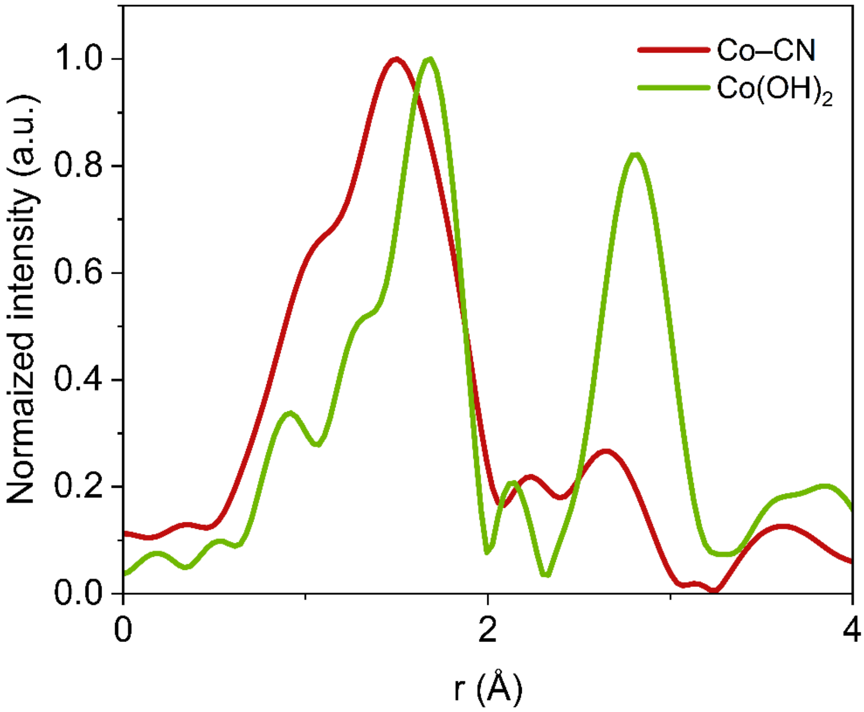

Figure S4. FT-EXAFS normalized spectra at Co K edge of Co–CN (red) and of $Co(OH)_2$ (green).

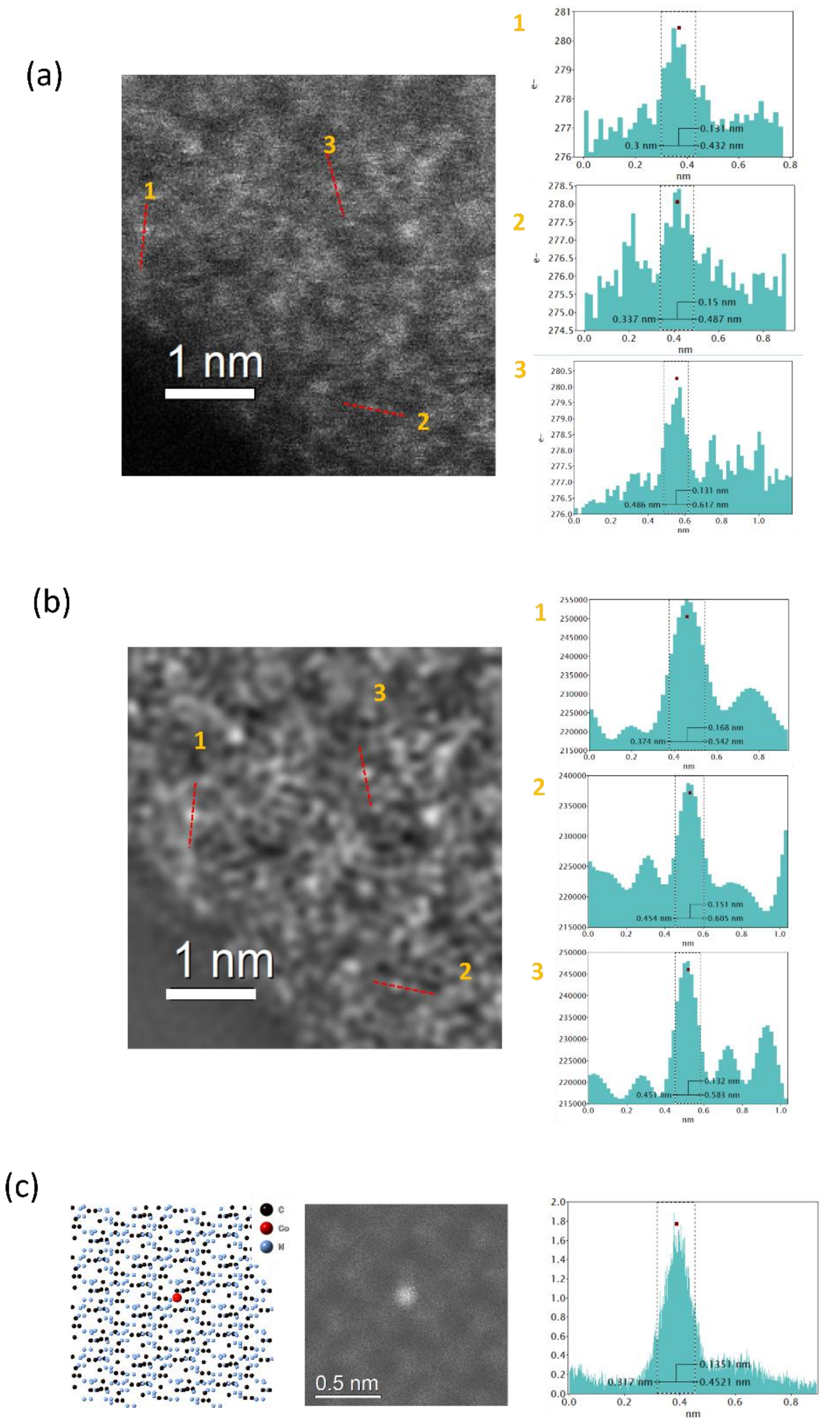

Figure S5. (a) Representative HAADF/STEM image of Co–CN with corresponding intensity line profiles; (b) FFT filtered HAADF/STEM image of Co–CN with corresponding intensity line profiles; (c) simulated image of Co–CN with poisson noise with corresponding intensity line profile.

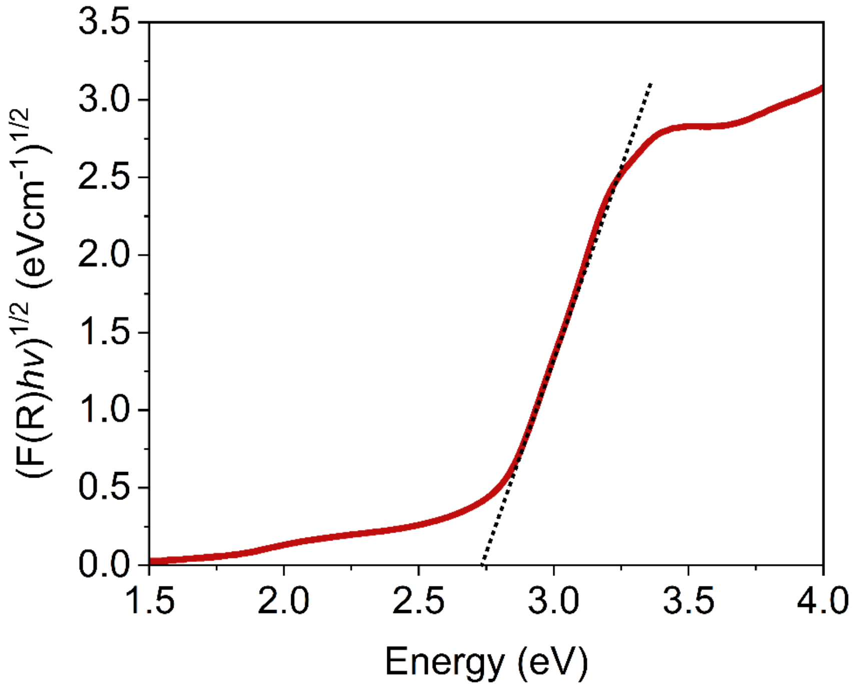

Figure S6. Tauc plot of Co–CN.

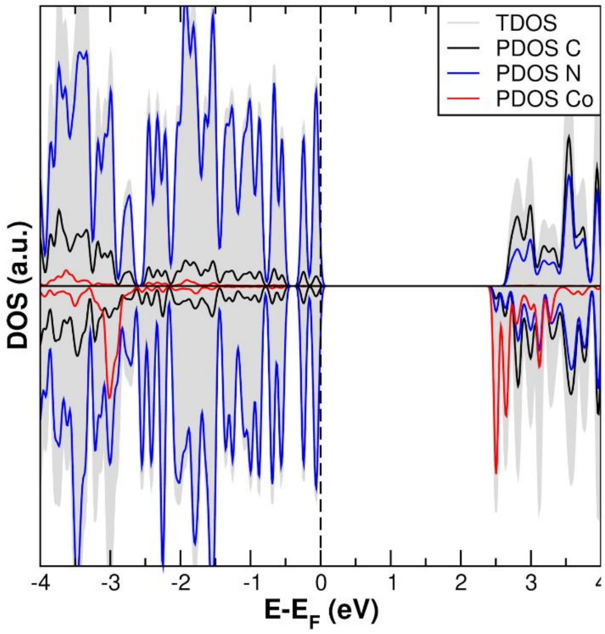

Figure S7. Total and projected density of states (DOS) for Co^{2+}–CN, as obtained from CRY17/HSE06+D3 calculations. The color legend is shown in the panel. The Fermi level is aligned to zero and indicated by a dashed line. The spin-up and spin-down components are plotted separately for direct comparison with the open-shell Co^{2+}–CN system. Spin-up and spin-down components are shown in the upper and lower sections of each plot, respectively.

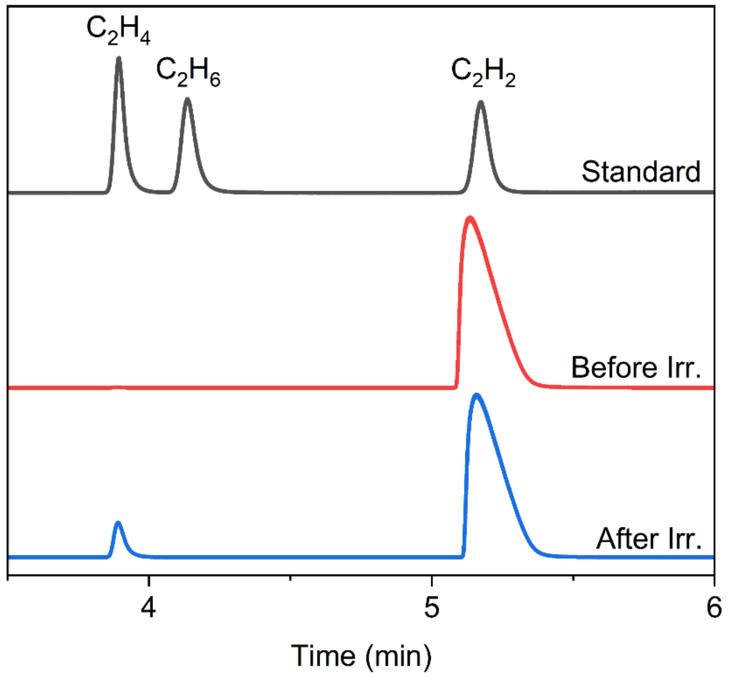

Figure S8. Typical gas chromatographs observed for the photoreduction of acetylene (retention time of C_2H_4, C_2H_6 and C_2H_2) detected with flame ion detection of a gas standard mixture (black) containing C_2H_4 (2.5 vol.%), C_2H_6 (2.5 vol.%) and C_2H_2 (2.5 vol.%) and the optimized system under C_2H_2 before (red) and after (blue) irradiation (405 nm, 140 mW·cm^{-2}) for 80 h. The optimized system contained 2.00 ± 0.05 mg Co–CN and 2.0 M TEOA in pure water at pH = 10.3 ± 0.1.

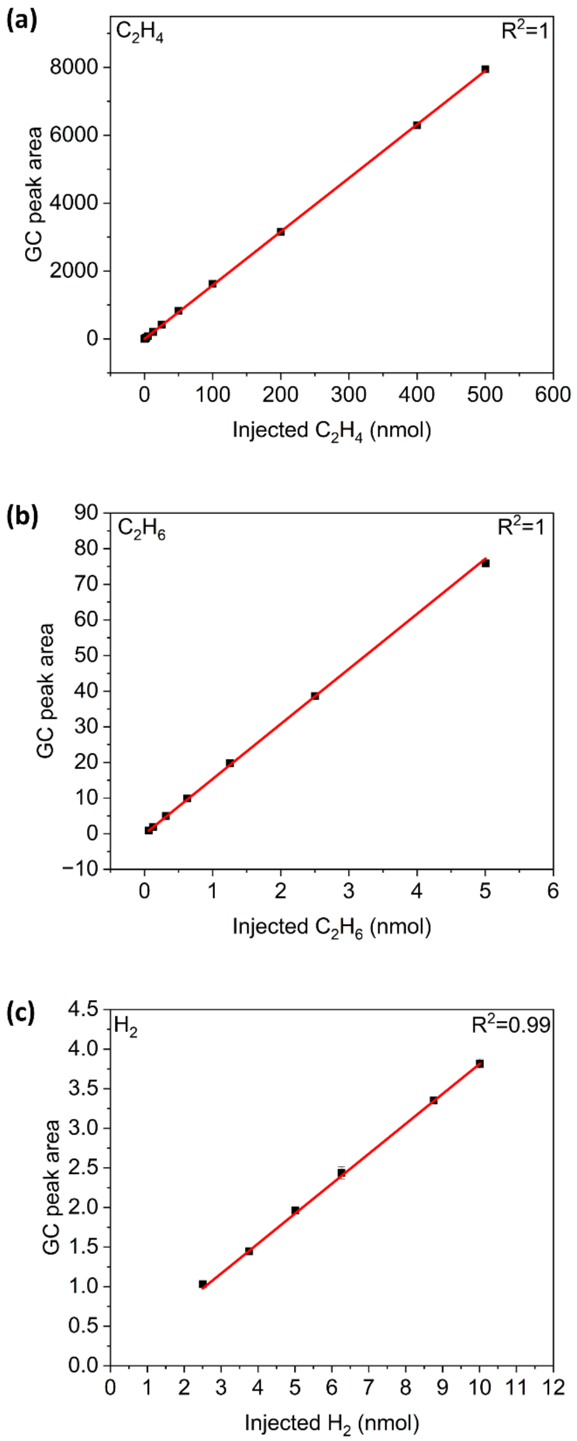

Figure S9. Calibration curves for quantification of gaseous products of the photoreduction of acetylene using GC-FID. (a) Calibration curve for C_2H_4 with the corresponding coefficient of linear correlation (R^2). (b) Calibration curve for C_2H_6 with the corresponding coefficient of linear correlation (R^2). (c) Calibration curve for H_2 with the corresponding coefficient of linear correlation (R^2). Error bars are calculated from three to four runs; uncertainty is ≤10%.

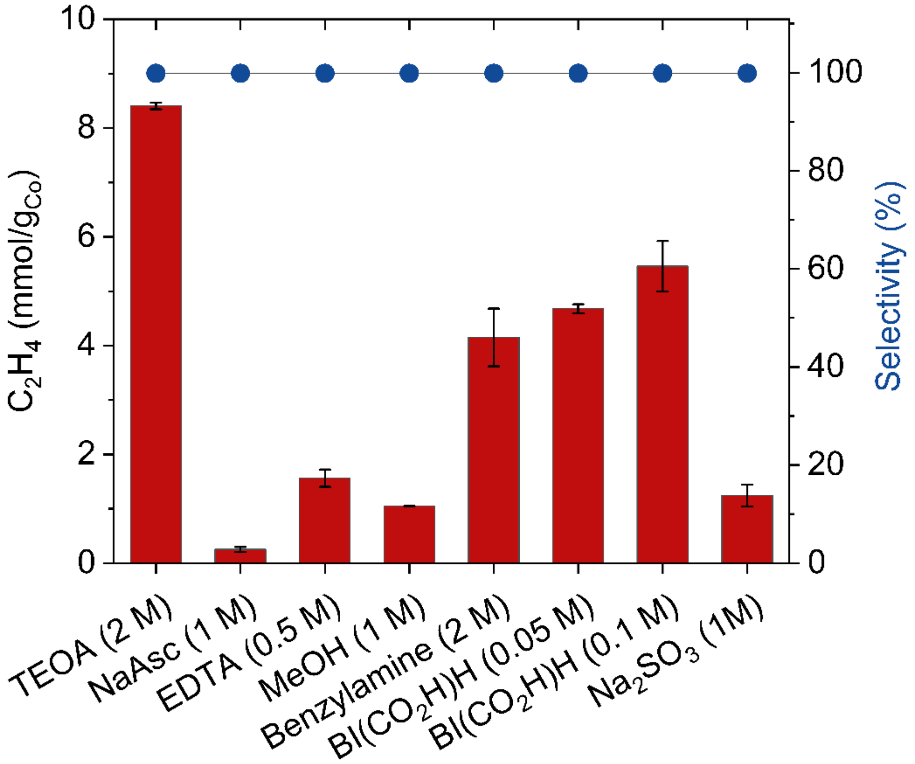

Figure S10. Photocatalytic ethylene production (left, red bar) and ethylene selectivity over both ethane and hydrogen (right, blue dots) through variation of the sacrificial donor. Optimization of amount of C_2H_4 produced through variation of the sacrificial donor in the presence of Co−CN (2.00 ± 0.05 mg) in water under C_2H_2 after irradiation with 405 nm light (140 mW·cm^{-2}) for 4 h. Error bars are calculated from two to three runs; uncertainty is ≤10%.

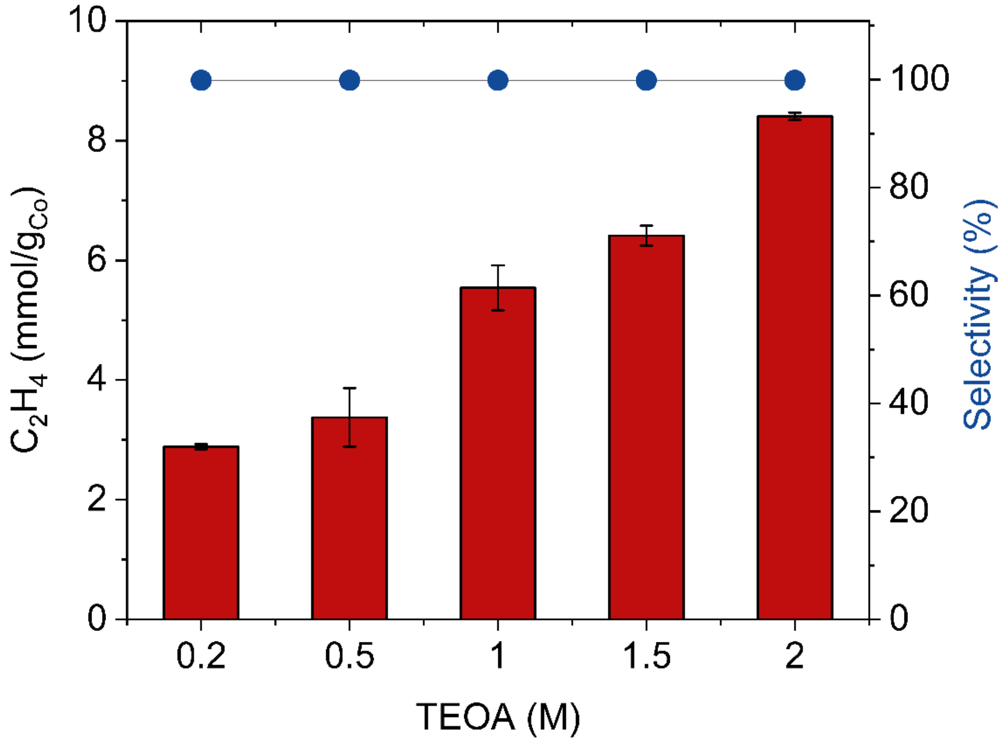

Figure S11. Photocatalytic ethylene production (left, red bar) and ethylene selectivity over both ethane and hydrogen (right, blue dots) through variation of TEOA concentration. Optimization of amount of C_2H_4 produced through variation of [TEOA] in the presence of Co−CN (2.00 ± 0.05 mg) in water under C_2H_2 after irradiation with 405 nm light (140 mW·cm^{-2}) for 4 h. Error bars are calculated from two to three runs; uncertainty is ≤10%.

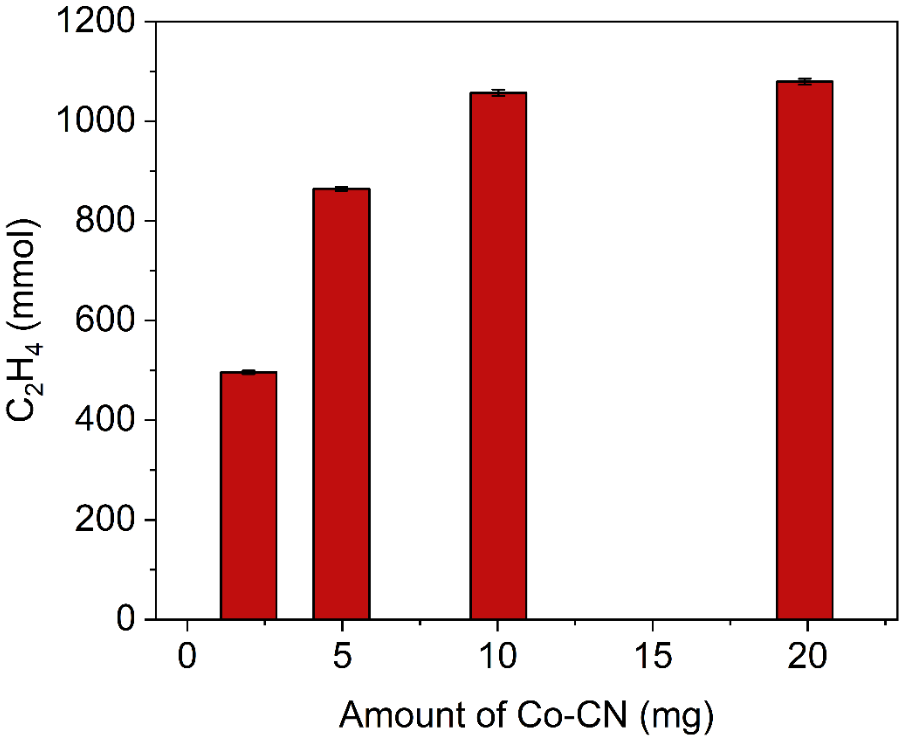

Figure S12. Photocatalytic ethylene production through variation of Co−CN amount. Optimization of amount of C_2H_4 produced through variation of Co−CN amount in the presence of 2.0 M TEOA in water under C_2H_2 after irradiation with 405 nm (140 mW·cm^{-2}) for 18 h. Error bars are calculated from two to three runs; uncertainty is ≤10%.

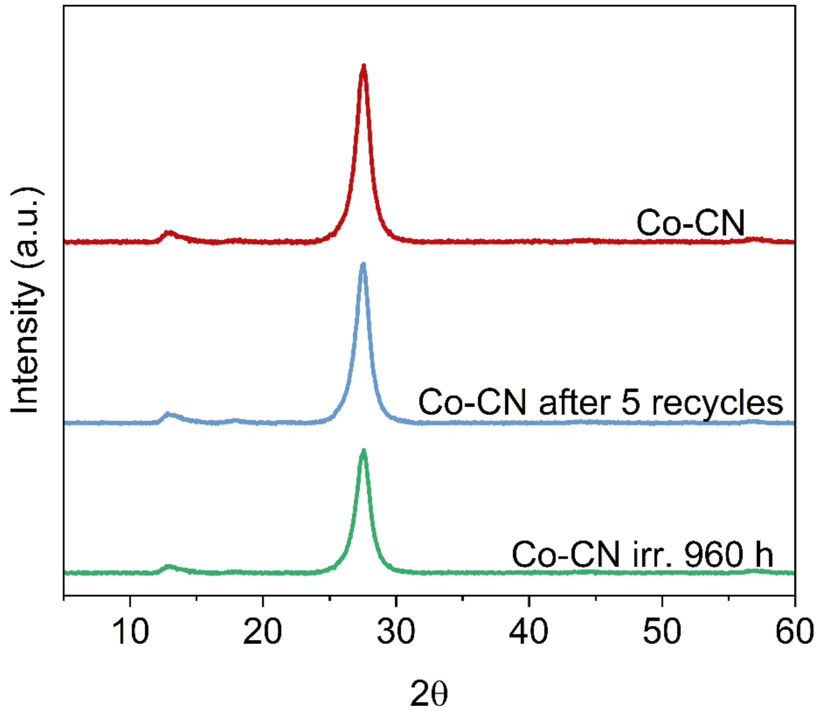

Figure S13. PXRD patterns of Co−CN powder (red), Co–CN after 5 cycles (blue), and Co–CN after 960 h of irradiation (post-catalysis, green).

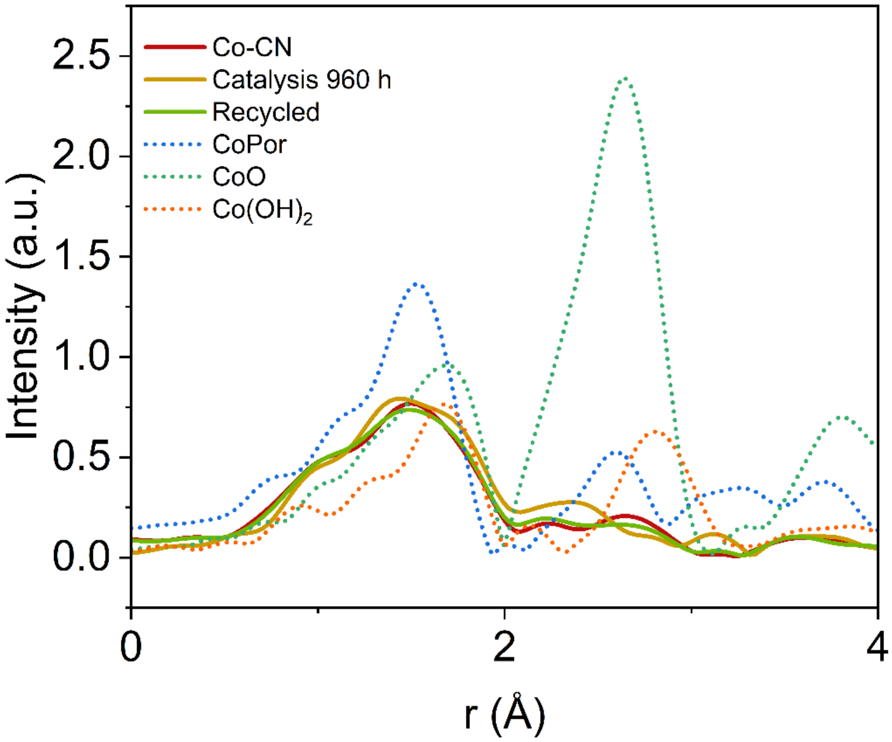

Figure S14. EXAFS spectra at the Co K edge of Co–CN powder (red), Co–CN powder recovered after 960 h of irradiation (yellow, post-catalysis, irradiation at 405 nm, 140 mW·cm^{-2} in the presence of 2.0 M TEOA, in pure water at pH = 10.3 ± 0.1, under C_2H_2), and the Co−CN powder recovered from recycling experiments (green, 5 cycles of 4 h irradiation each, 405 nm, 140 mW·cm^{-2} in the presence of 2.0 M TEOA, pure water at pH = 10.3 ± 0.1, under C_2H_2). EXAFS of Co-porphyrin (CoTPP) (blue dots), CoO (green dots), and $Co(OH)_2$ (orange dots) as references.

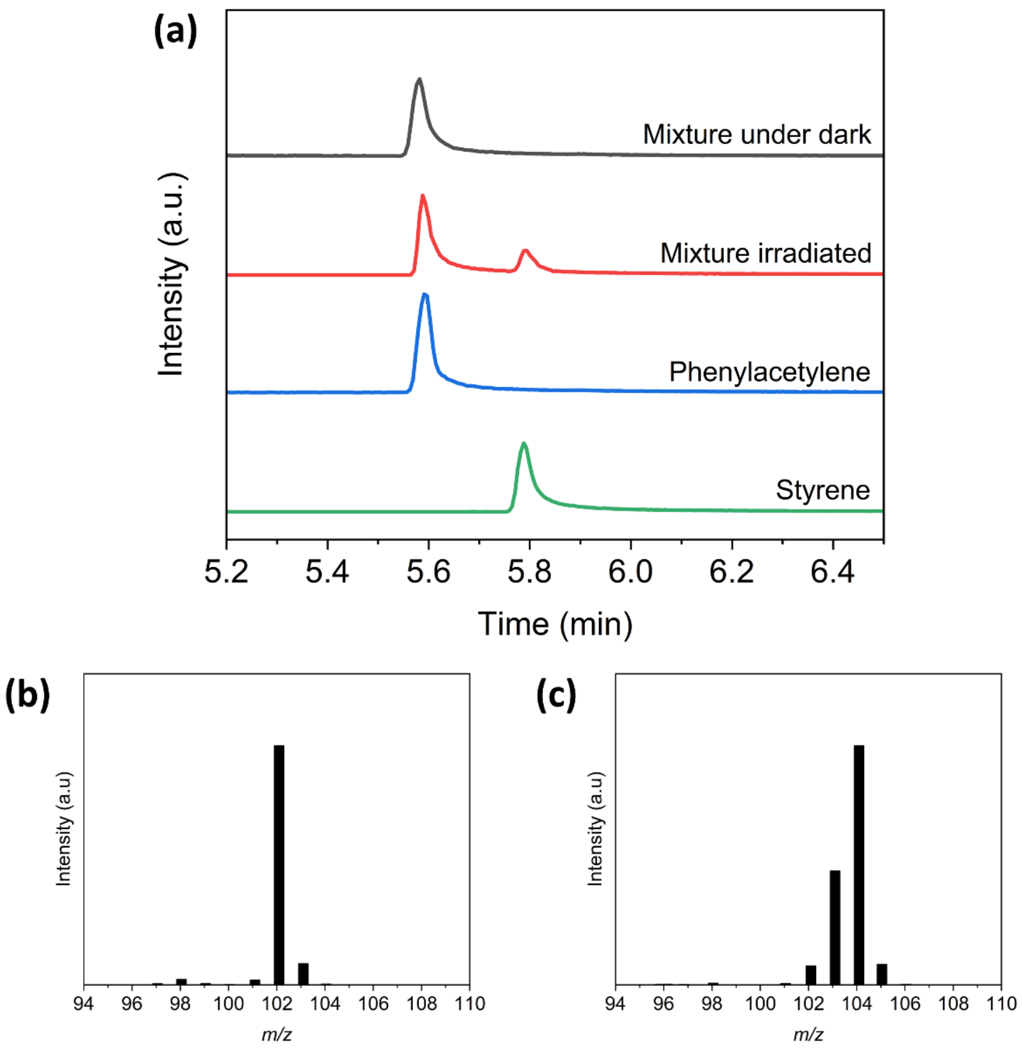

Figure S15. GC-MS chromatograms and mass spectra observed for the photoreduction reaction of phenylacetylene. (a) Gas chromatograms of an acetonitrile solution containing 2.00 ± 0.05 mg Co−CN, 10 mM phenylacetylene and 1.0 M TEOA kept in the dark (black) or after irradiation (red) for 24 h (405 nm, 140 mW·cm^{-2}). Phenylacetylene standard (blue) and styrene standard (green). (b) Mass spectrum of phenylacetylene. (c) Mass spectrum of styrene.

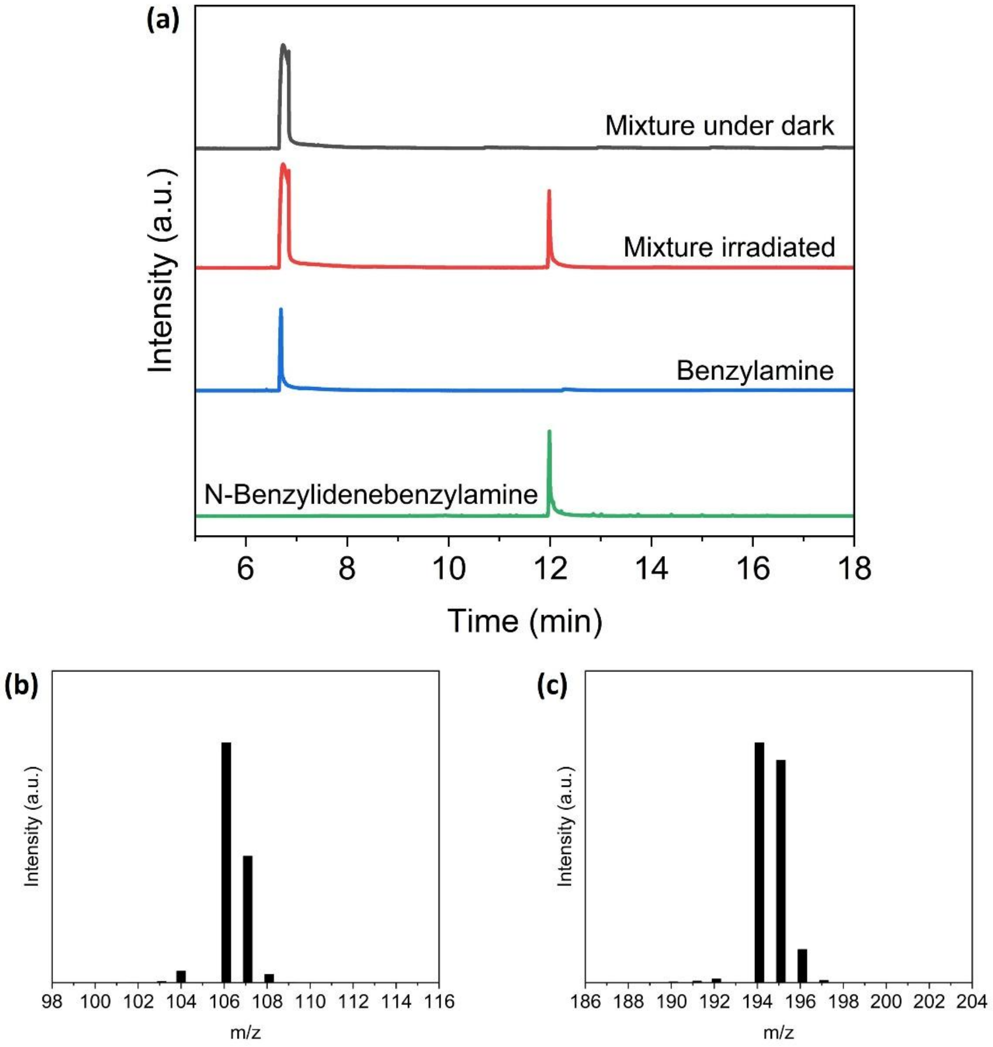

Figure S16. GC-MS chromatograms and mass spectra observed for the photoreduction reaction of C_2H_2 in the presence of 2.0 M benzylamine using 2.00 ± 0.05 mg of Co−CN. (a) Gas chromatograms of a solution containing benzylamine kept in the dark (black) or after irradiation (red) for 90 h (405 nm, 140 mW·cm^{-2}). Benzylamine standard (blue) and *N*-benzylidenebenzylamine standard (green). (b) Mass spectrum of benzylamine. (c) Mass spectrum of *N*-benzylidenebenzylamine.

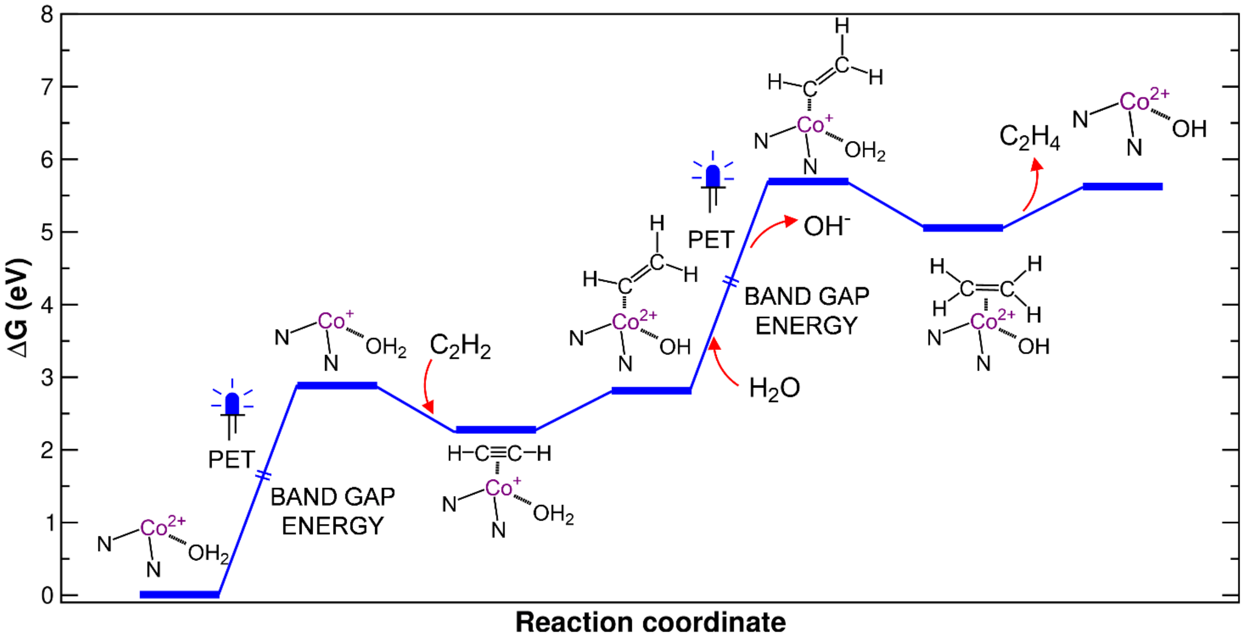

Figure S17. Free energy profile for the hydrogenation of C_2H_2 to C_2H_4 catalyzed by Co–CN, as computed using QE/PBE+U+D3. The energy increases observed during the two photoinduced electron transfer (PET) steps correspond approximately to the calculated band gap of the CN material (2.88 eV), as obtained from CRY17/HSE06+D3 calculations.

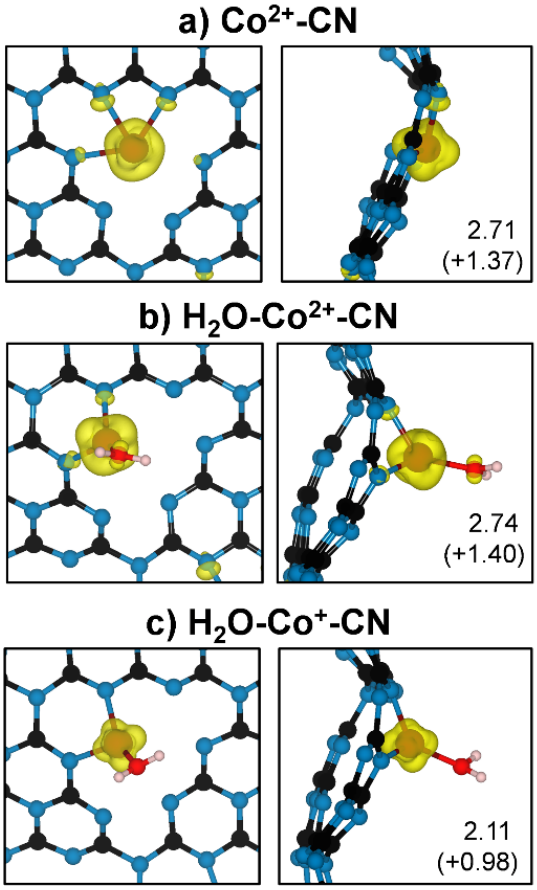

Figure S18. Isosurface 3D plots (top and side views) of the spin density distribution (ρ_α-ρ_β) for a) Co^{2+}–CN, b) H_2O–Co^{2+}–CN, and c) H_2O–Co^{+}–CN, as calculated using QE/PBE+U+D3. Systems (b) and (c) correspond to the first two intermediates in the reaction pathway shown in Figure S17. The isosurface value is set to 1×10^{-2} e^-/bohr^3. Numbers in the right panel represent the spin polarization on the Co atom, with the corresponding atomic charge (in parentheses) calculated using Bader charge analysis.

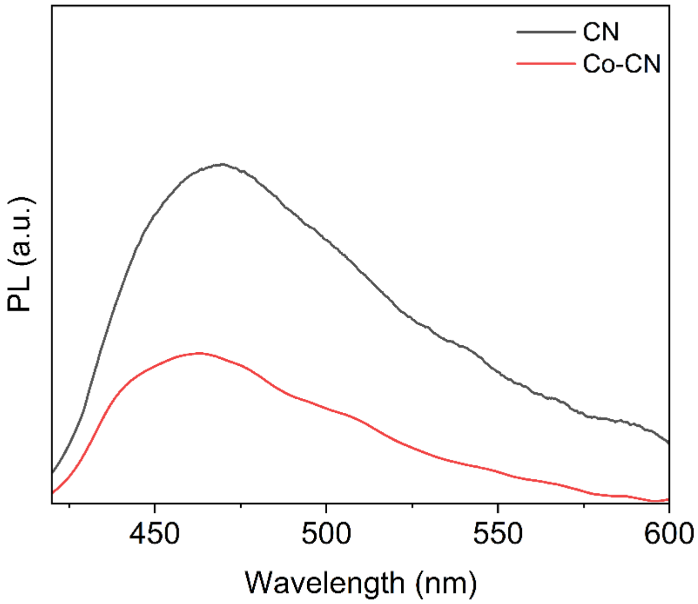

Figure S19. Solid steady-state photoluminescence of CN and Co–CN.

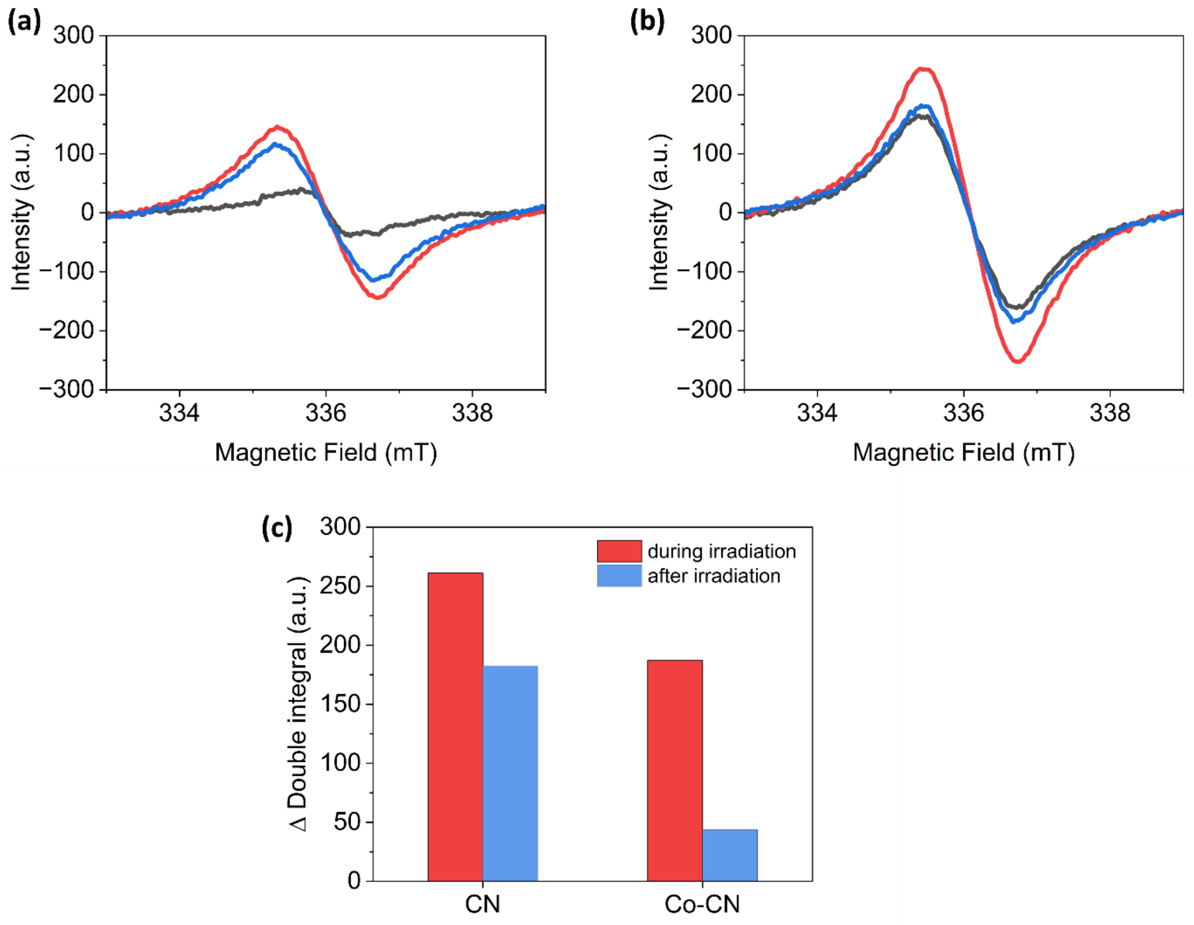

Figure S20. (a) EPR spectra of pristine CN powder at rt before irradiation (black), during visible light (405 nm, 140 mW·cm^{-2}) irradiation (red) and 10 min after switching off the light (blue). (b) EPR spectra of Co–CN powder at rt before irradiation (black), during visible light (405 nm, 140 mW·cm^{-2}) irradiation (red) and 10 min after switching off the light (blue). (c) Difference between the double integral of the EPR signals before irradiation (red) and after irradiation (blue).

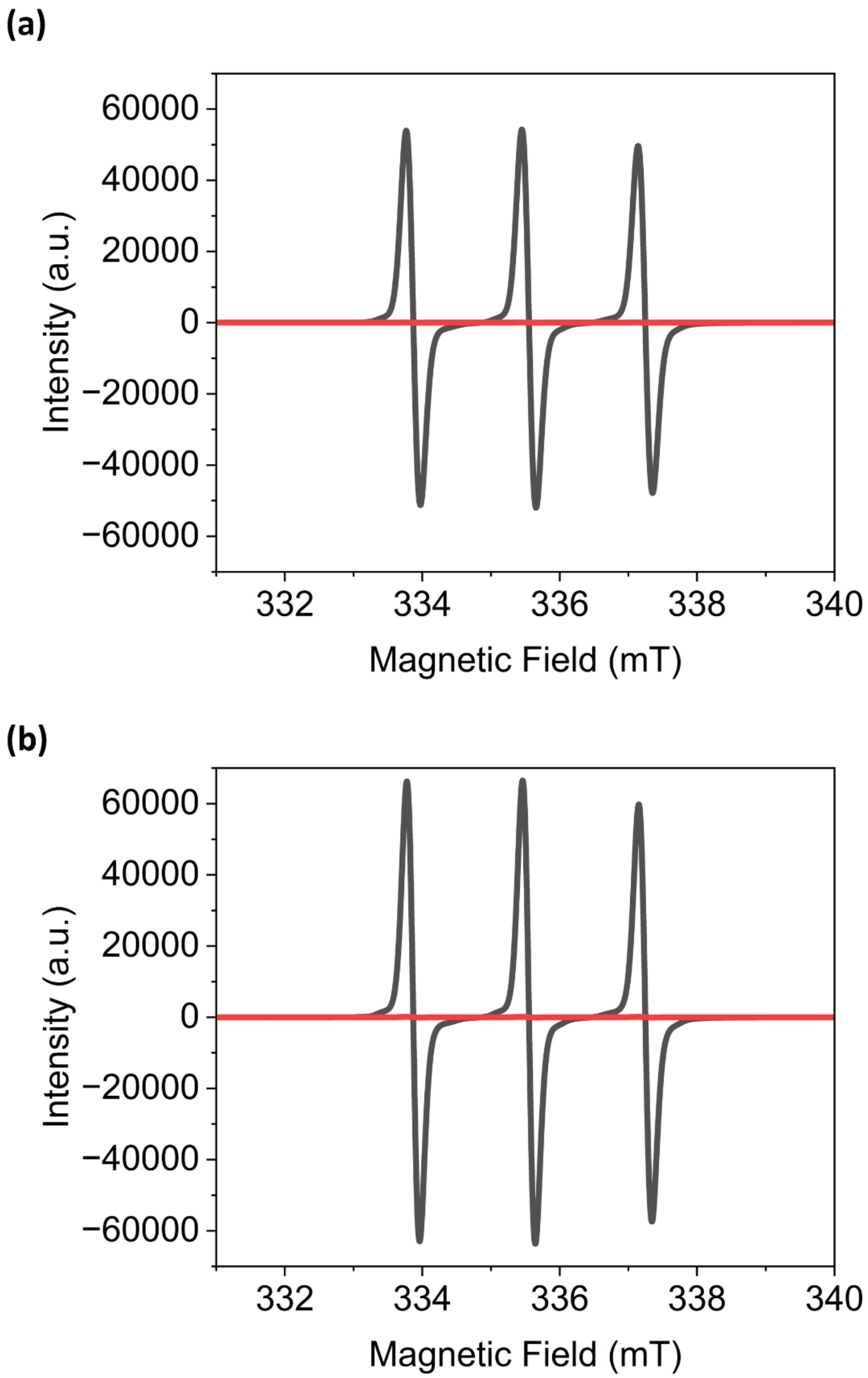

Figure S21. (a) EPR spectra of Co–CN (2.00 ± 0.05 mg), TEOA (2.0 M), TEMPO (1 mg) in pure water at pH = 10.3 ± 0.1 under C_2H_2 atmosphere, under dark (black) or irradiated for 4 h at 405 nm (140 mW·cm^{-2}). (b) EPR spectra of Co–CN (2.00 ± 0.05 mg), TEOA (2.0 M), TEMPO (1 mg) in pure water at pH = 10.3 ± 0.1 under Ar atmosphere, under dark (black) or irradiated for 4 h at 405 nm (140 mW·cm^{-2}).

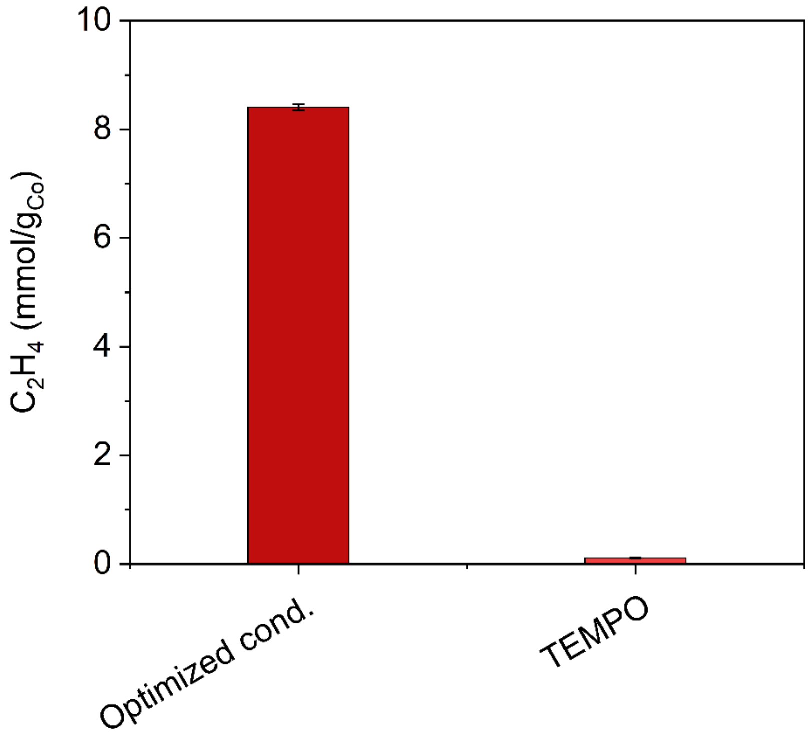

Figure S22. Comparison of the photocatalytic ethylene production in 4 h irradiation (405 nm, 140 mW·cm^{-2}) by the Co−CN under the optimized conditions or in the presence of 200 equiv. of TEMPO *vs* Co. The optimized system is a C_2H_2-purged solution containing 2.00 ± 0.05 mg of Co−CN, 2.0 M TEOA in pure water at pH = 10.3 ± 0.1.

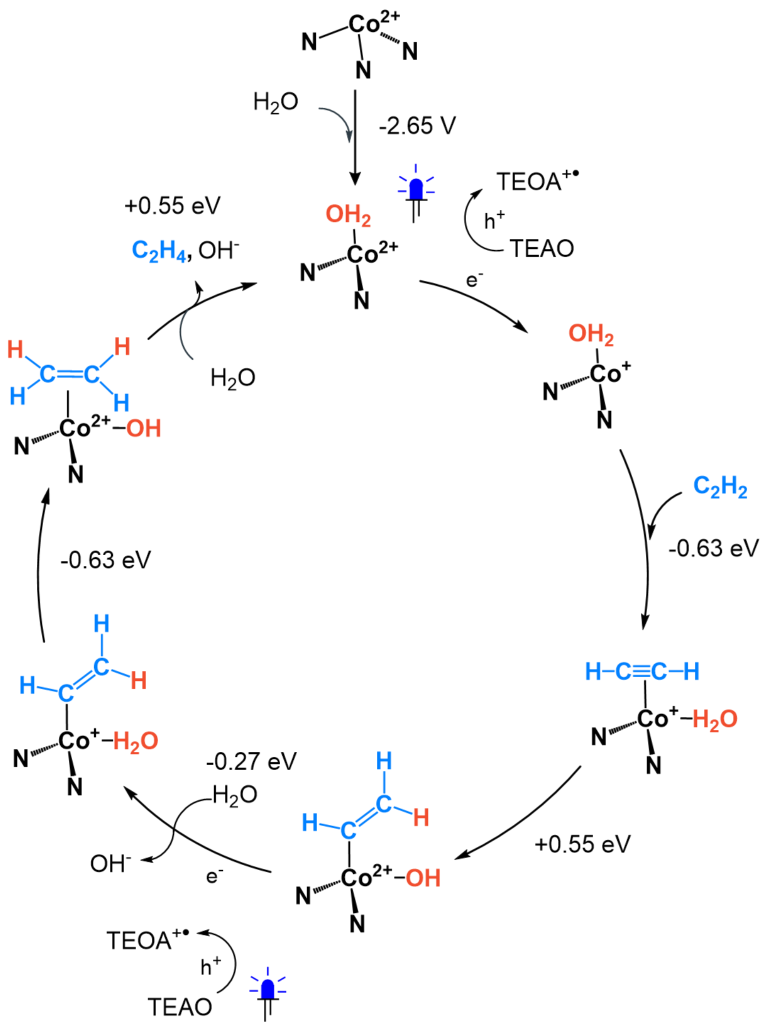

Figure S23. The proposed visible-light driven semi-hydrogenation mechanism for ethylene production in water mediated by Co–CN, based on DFT/PBE+U+D3 calculations. The Co single atom is in the 2+ oxidation state and is coordinated to three pyridinic N atoms, as shown in Figure S3. Upon binding of one water molecule, the Co center loses one coordination with pyridinic N. The photoexcitation process is modelled by adding one extra electron to the system. The Co charged state is determined by the atomic spin and the Bader charges. When the total charge is not varied, we can compute Gibbs free energy variations (ΔG in eV) which are reported in the scheme.

The two protons added to make the ethylene product are labeled in red to better visualize the proton transfer steps.

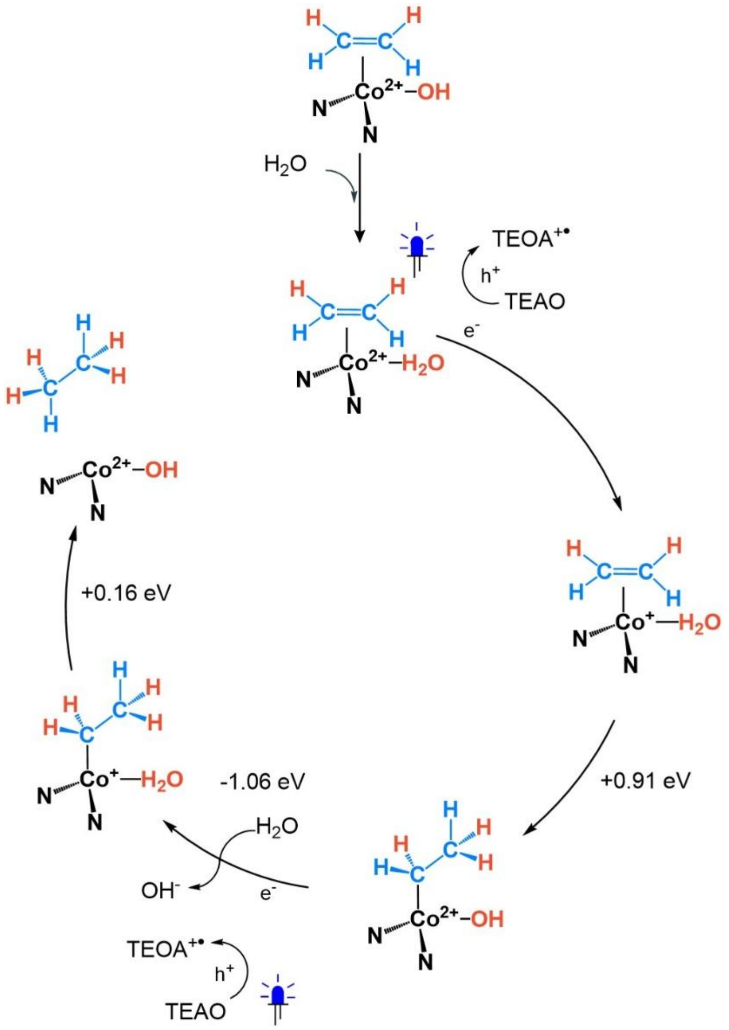

Figure S24. The proposed visible-light-driven hydrogenation mechanism for ethane production in water, mediated by Co–CN, starts from the final intermediate of the cycle shown in Figure S23. Initially, a water molecule replaces the OH^- bound to Co^{2+}, restoring the proton source necessary for ethylene protonation. The photoexcitation process is modeled by introducing an extra electron into the system. The charge state of Co is determined through atomic spin analysis and Bader charge calculations. When the total charge remains unchanged, Gibbs free energy variations (ΔG in eV) are computed and reported in the scheme.

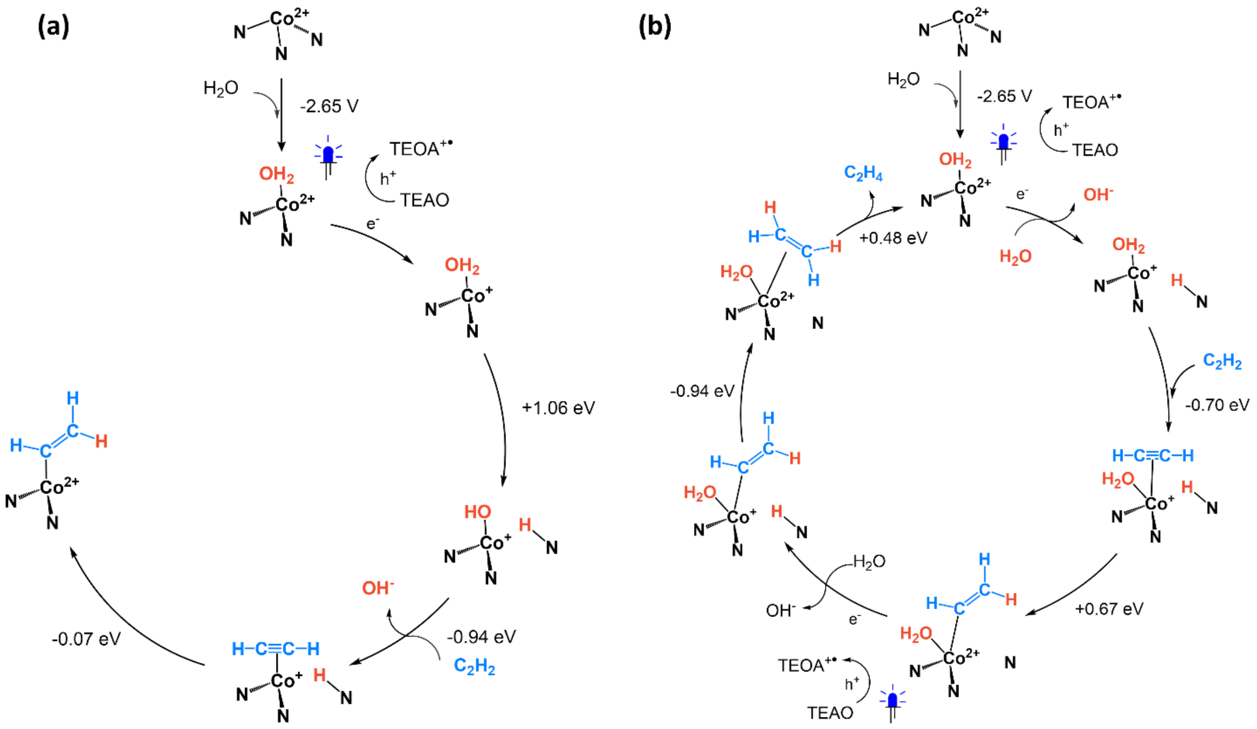

Figure S25. Two alternative visible-light-driven semi-hydrogenation mechanisms for ethylene production in water, mediated by Co–CN, proceeding through proton transfer from a proximal protonated pyridinic N. In the first cycle (a) water dissociation occurs, leading to the binding of the OH^- fragment to Co^+ and the protonation of a nearby pyridinic N. This is followed by acetylene adsorption on the metal center, replacing the OH^- ligand. Conversely, in the second cycle (b), protonation of a pyridinic N is produced by a water molecule in solution, while the initially adsorbed water molecule remains coordinated to the metal throughout the reaction.

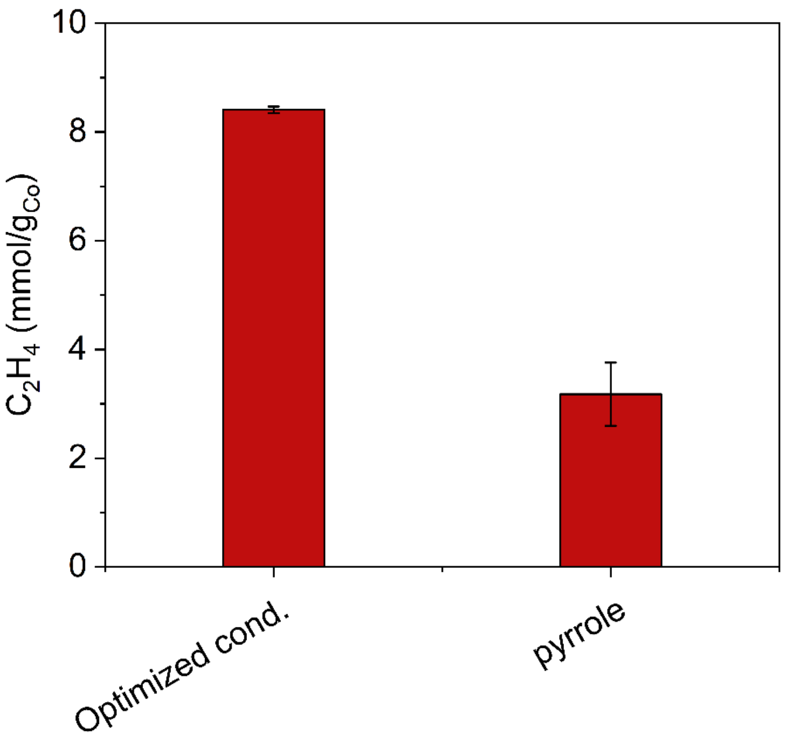

Figure S26. Comparison of the photocatalytic ethylene production in 4 h irradiation (405 nm, 140 mW·cm^{-2}) by the Co−CN under the optimized conditions or in the presence of 200 equiv. of pyrrole *vs* Co. The optimized system contained 2.00 ± 0.05 mg of Co−CN, 2.0 M TEOA in pure water at pH = 10.3 ± 0.1.

Supplementary Tables

Table S1. Analysis of the C 1*s* XPS region for the Co–CN sample. For each component, the binding energy and the percentage are reported.

C–C (%)	C–O (%)	N–C=N (%)
284.7 eV (43.6%)	286.2 eV (9.1%)	288.3 eV (47.3%)

Table S2. Analysis of the N 1*s* XPS region for the Co–CN sample. For each component, the binding energy and the percentage are reported.

C–N=C (%)	(C_2)–NH (%)	$(C)_3$–N (%)
398.7 eV (67.9%)	399.9 eV (20.5%)	401.15 eV (11.6%)

Table S3. Structural parameters obtained for the Co–CN sample from fitting the Co K edge FT EXAFS data (k = 2.3 – 12; R = 1.0 – 3.2 Å). c.n. is the coordination number, R is the interatomic distance, and σ^2 is the Debye-Waller factor.

Shell	c.n.	R(Å)	σ^2 x 10^4 ($Å^2$)
Co-N_1	2.0	2.02±0.02	42.6±13.3
Co-N_2	1.0	2.17±0.07	42.7±8.5

Table S4. ICP quantification.

Sample	Co (% w/w)
Co–CN	1.00 ± 0.05
Co–CN after 960 h of photocatalysis	0.98 ± 0.05
Supernatant after 960 h of photocatalysis	<LoD
Co–CN after five photocatalytic cycles of 4 h each	1.00 ± 0.05

Note: the Co content of the photocatalyst well compare with other cobalt single-atom catalysts supported on carbon nitride[17, 18] *vs* the Ni content reported in the literature procedure that was adapted for this work.[3]

Table S5. The comparison of catalytic performance with state-of-the-art photocatalytic systems for reduction of C_2H_2 to C_2H_4 under pure acetylene atmosphere.

Photocat. mixture	C_2H_4 Production	Sel_{C2H4} (%)	Longevity/ irr. time	Recyclability	Reference
CoTPPS (Hom. Cat.) $[Ru(bpy)_3]^{2+}$ (PS) NaAsc (SD) Bicarbonate buffer (H_2O PD)	3.93 μmol	99.4	24 h	n.d.	*Nat. Chem.* **2022**, *12*, 1007-1012
Co-PCN-222 (Het. Cat.) $[Ru(bpy)_3]^{2+}$ (PS) TEOA (SD and PD) ACN	160 mmol/g_{Co}	99.7	168 h	5 cycles	*ACS Energy Lett.* **2023**, 8, *11*, 4684-4693
Cu-Co-MNSs (Het. Cat.) $[Ru(bpy)_3]^{2+}$ (PS) NaAsc (SD) Bicarbonate buffer (H_2O PD)	3.61 mmol/g_{Co}	99.5	12 h	n.d	*Chem. Eur. J.* **2024**, *30*, e202302816
$Co(dmgH)_2pyCl$ (Hom. Cat.) $[Ru(bpy)_3]^{2+}$ (PS) BIH (SD) TFE (PD) ACN	63 μmol	99.9	20 h	n.d.	*Adv. Mater*. **2025**, *37*, 2408658
Cz-Co-COF-H (Het. Cat.) $[Ru(bpy)_3]^{2+}$ (PS) NaAsc (SD) H_2O (PD)	1.7 mmol/(g $_{Co}$ h)	99.9	12 h	5 cycles	*Angew. Chem. Int. Ed.* **2025**, *64*, e202423091
$[Co^{II}(N_4S_2)](ClO_4)_2$ (Hom. Cat.) $[Cu(L1)(L2)]PF_6$ (PS) NaAsc (SD) Bicarbonate buffer (H_2O PD)	16.15 μmol^{+}	97.1	15 h	n.d.	*ACS Catal*. **2025**, *15*, 1135-1146
Co-CN (Het. Cat. and PS) TEOA (SD) H_2O (PD)	390 mmol/g_{Co}	99.9	> 960 h	5 cycles	This work

PS=photosensitizer, SD=sacrificial donor; PD=proton donor, L1=sodium 2,9-dimethyl-4,7-diphenyl-1,10-phenanthroline-5,6-disulfonate, L2=9,9-dimethyl-2,7-bissulfonato-4,5-bis(diphenylphosphino)xanthene sodium salt. C_2H_4 production refers to the highest reported irradiation time/longevity of the system. Sel_{C2H4} refers to the ethylene selectivity over ethane.

$^{+}$under 1% C_2H_2 (Ar balanced).

Supplementary References

1. Arcudi, F.; Đorđević, L.; Schweitzer, N.; Stupp, S. I.; Weiss, E. A., Selective visible-light photocatalysis of acetylene to ethylene using a cobalt molecular catalyst and water as a proton source, *Nat. Chem.* **2022,** *14* (9), 1007-1012. DOI 10.1038/s41557-022-00966-5.
2. Wang, Y.-F.; Zhang, M.-T., Proton-coupled electron-transfer reduction of dioxygen: the importance of precursor complex formation between electron donor and proton donor, *J. Am. Chem. Soc.* **2022,** *144* (27), 12459-12468. DOI 10.1021/jacs.2c04467.
3. Vilé, G.; Sharma, P.; Nachtegaal, M.; Tollini, F.; Moscatelli, D.; Sroka-Bartnicka, A.; Tomanec, O.; Petr, M.; Filip, J.; Pieta, I. S., An earth-abundant Ni-based single-atom catalyst for selective photodegradation of pollutants, *Solar RRL* **2021,** *5* (7), 2100176-2100188. DOI 10.1002/solr.202100176.
4. Giannozzi, P.; Baroni, S.; Bonini, N.; Calandra, M.; Car, R.; Cavazzoni, C.; Ceresoli, D.; Chiarotti, G. L.; Cococcioni, M.; Dabo, I., QUANTUM ESPRESSO: a modular and open-source software project for quantumsimulations of materials, *J. Phys.: Condens. Matter* **2009,** *21* (39), 395502. DOI 10.1088/0953-8984/21/39/395502.
5. Giannozzi, P.; Andreussi, O.; Brumme, T.; Bunau, O.; Nardelli, M. B.; Calandra, M.; Car, R.; Cavazzoni, C.; Ceresoli, D.; Cococcioni, M., Advanced capabilities for materials modelling with Quantum ESPRESSO, *J. Phys.: Condens. Matter* **2017,** *29* (46), 465901. DOI 10.1088/1361-648X/aa8f79.
6. Dovesi, R.; Saunders, V.; Roetti, C.; Orlando, R.; Zicovich-Wilson, C.; Pascale, F.; Civalleri, B.; Doll, K.; Harrison, N.; Bush, I., Crystal 17, **2017**.
7. Dal Corso, A., Pseudopotentials periodic table: From H to Pu, *Comput. Mater. Sci.* **2014,** *95*, 337-350. DOI 10.1016/j.commatsci.2014.07.043.
8. Grimme, S.; Antony, J.; Ehrlich, S.; Krieg, H., Communications: Explicitly correlated second-order Møller–Plesset perturbation method for extended systems, *J. Chem. Phys.* **2010,** *132* (15). DOI 10.1063/1.3396079.
9. Himmetoglu, B.; Floris, A.; De Gironcoli, S.; Cococcioni, M., Hubbard-corrected DFT energy functionals: The LDA+ U description of correlated systems, *Int. J. Quantum Chem* **2014,** *114* (1), 14-49. DOI 10.1002/qua.24521.
10. Krukau, A. V.; Vydrov, O. A.; Izmaylov, A. F.; Scuseria, G. E., Influence of the exchange screening parameter on the performance of screened hybrid functionals, *J. Chem. Phys.* **2006,** *125* (22). DOI 10.1063/1.2404663.
11. Vilela Oliveira, D.; Laun, J.; Peintinger, M. F.; Bredow, T., BSSE-correction scheme for consistent gaussian basis sets of double-and triple-zeta valence with polarization quality for solid-state calculations, *J. Comput. Chem.* **2019,** *40* (27), 2364-2376. DOI 10.1002/jcc.26013
12. Ugolotti, A.; Di Valentin, C., Ab-initio spectroscopic characterization of melem-based graphitic carbon nitride polymorphs, *Nanomaterials* **2021,** *11* (7), 1863. DOI 10.3390/nano11071863.
13. Henkelman, G.; Arnaldsson, A.; Jónsson, H., A fast and robust algorithm for Bader decomposition of charge density, *Comput. Mater. Sci.* **2006,** *36* (3), 354-360. DOI 10.1016/j.commatsci.2005.04.010.
14. Monkhorst, H. J.; Pack, J. D., Special points for Brillouin-zone integrations, *Phys. Rev. B* **1976,** *13* (12), 5188. DOI 10.1103/PhysRevB.13.5188
15. Rossmeisl, J.; Logadottir, A.; Nørskov, J. K., Electrolysis of water on (oxidized) metal surfaces, *Chem. Phys.* **2005,** *319* (1-3), 178-184. DOI 10.1016/j.chemphys.2005.05.038
16. Valdés, Á.; Qu, Z.-W.; Kroes, G.-J.; Rossmeisl, J.; Nørskov, J. K., Oxidation and Photo-Oxidation of Water on TiO_2 Surface, *J. Phys. Chem. C* **2008,** *112* (26), 9872-9879. DOI 10.1021/jp711929d

17. Fu, J.; Zhu, L.; Jiang, K.; Liu, K.; Wang, Z.; Qiu, X.; Li, H.; Hu, J.; Pan, H.; Lu, Y.-R., Activation of CO_2 on graphitic carbon nitride supported single-atom cobalt sites, *Chem. Eng. J.* **2021,** *415*, 128982. DOI 10.1016/j.cej.2021.128982
18. Cao, Y.; Chen, S.; Luo, Q.; Yan, H.; Lin, Y.; Liu, W.; Cao, L.; Lu, J.; Yang, J.; Yao, T., Atomic-Level Insight into Optimizing the Hydrogen Evolution Pathway over a Co_1-N_4 Single-Site Photocatalyst, *Angew. Chem. Int. Ed.* **2017,** *56* (40), 12191-12196. DOI 10.1002/anie.201706467.
